# Supplementary material for: Quality of aerobic training description and its relation to intervention efficacy in chronic obstructive pulmonary disease trials: study protocol for a systematic review, meta-analysis and meta-regression
Source: BMJ Open. 2024 May 24;14(5):e084296. doi: 10.1136/bmjopen-2024-084296 (PMC11129019; doi:10.1136/bmjopen-2024-084296)
Supplement: Supplementary data [file bmjopen-2024-084296supp001.pdf]

Reporting checklist for protocol of a systematic review and meta analysis.

Based on the PRISMA-P guidelines.

Instructions to authors

Complete this checklist by entering the page numbers from your manuscript where readers will find each of the items listed below.

Your article may not currently address all the items on the checklist. Please modify your text to include the missing information. If you are certain that an item does not apply, please write "n/a" and provide a short explanation.

Upload your completed checklist as an extra file when you submit to a journal.

In your methods section, say that you used the PRISMA-Preporting guidelines, and cite them as:

Moher D, Shamseer L, Clarke M, Ghersi D, Liberati A, Petticrew M, Shekelle P, Stewart LA. Preferred Reporting Items for Systematic Review and Meta-Analysis Protocols (PRISMA-P) 2015 statement. Syst Rev. 2015;4(1):1.

		Reporting Item	Page Number
**Title**			
Identification	#1a	Identify the report as a protocol of a systematic review	1
Update	#1b	If the protocol is for an update of a previous systematic review, identify as such	NA
**Registration**			
	#2	If registered, provide the name of the registry (such as PROSPERO) and registration number	2
**Authors**			
Contact	#3a	Provide name, institutional affiliation, e-mail address of all protocol authors; provide physical mailing address of corresponding author	1
Contribution	#3b	Describe contributions of protocol authors and identify the	17

		guarantor of the review	
**Amendments**			
	#4	If the protocol represents an amendment of a previously completed or published protocol, identify as such and list changes; otherwise, state plan for documenting important protocol amendments	16
**Support**			
Sources	#5a	Indicate sources of financial or other support for the review	17
Sponsor	#5b	Provide name for the review funder and / or sponsor	NA
Role of sponsor or funder	#5c	Describe roles of funder(s), sponsor(s), and / or institution(s), if any, in developing the protocol	17
**Introduction**			
Rationale	#6	Describe the rationale for the review in the context of what is already known	4-5
Objectives	#7	Provide an explicit statement of the question(s) the review will address with reference to participants, interventions, comparators, and outcomes (PICO)	5
**Methods**			
Eligibility criteria	#8	Specify the study characteristics (such as PICO, study design, setting, time frame) and report characteristics (such as years considered, language, publication status) to be used as criteria for eligibility for the review	5-6
Information sources	#9	Describe all intended information sources (such as electronic databases, contact with study authors, trial registers or other grey literature sources) with planned dates of coverage	6-7
Search strategy	#10	Present draft of search strategy to be used for at least one electronic database, including planned limits, such that it could be repeated	7 and appendix
Study records - data management	#11a	Describe the mechanism(s) that will be used to manage records and data throughout the review	7-8

Study records - selection process	#11b	State the process that will be used for selecting studies (such as two independent reviewers) through each phase of the review (that is, screening, eligibility and inclusion in meta-analysis)	7
Study records - data collection process	#11c	Describe planned method of extracting data from reports (such as piloting forms, done independently, in duplicate), any processes for obtaining and confirming data from investigators	8-12
Data items	#12	List and define all variables for which data will be sought (such as PICO items, funding sources), any pre-planned data assumptions and simplifications	8-12
Outcomes and prioritization	#13	List and define all outcomes for which data will be sought, including prioritization of main and additional outcomes, with rationale	12
Risk of bias in individual studies	#14	Describe anticipated methods for assessing risk of bias of individual studies, including whether this will be done at the outcome or study level, or both; state how this information will be used in data synthesis	12-13 + 15
Data synthesis	#15a	Describe criteria under which study data will be quantitatively synthesised	14-15
Data synthesis	#15b	If data are appropriate for quantitative synthesis, describe planned summary measures, methods of handling data and methods of combining data from studies, including any planned exploration of consistency (such as I2, Kendall's τ)	14-15
Data synthesis	#15c	Describe any proposed additional analyses (such as sensitivity or subgroup analyses, meta-regression)	14-15
Data synthesis	#15d	If quantitative synthesis is not appropriate, describe the type of summary planned	13-14
Meta-bias(es)	#16	Specify any planned assessment of meta-bias(es) (such as publication bias across studies, selective reporting within studies)	14
Confidence in cumulative evidence	#17	Describe how the strength of the body of evidence will be assessed (such as GRADE)	15-16

Notes:

- 10: 8 and appendix The PRISMA-P elaboration and explanation paper is distributed under the terms of the Creative Commons Attribution License CC-BY. This checklist was completed on 12. January 2024 using https://www.goodreports.org/, a tool made by the EQUATOR Network in collaboration with Penelope.ai

Begränsad delning

Online appendix 2. Search strategies

The number within brackets after every search terms is the number of hits for that search.

1. Search strategy for PubMed

Date: 27-05-2021

#1:

"Pulmonary Disease, Chronic Obstructive"[Mesh] OR Chronic Obstructive Pulmonary Disease* [tiab] OR COPD [tiab] OR COAD [tiab] OR Chronic Obstructive Airway Disease* [tiab] OR Chronic Obstructive Lung Disease* [tiab] OR chronic airflow obstruction* [tiab] OR chronic airflow limitation*[tiab] OR Emphysema[tiab] OR Chronic bronchitis[tiab] OR chronic obstructive bronchitis[tiab] OR chronic obstructive bronchopulmonary disease*[tiab] OR chronic obstructive lung disorder*[tiab] OR chronic obstructive pulmonary disorder*[tiab] OR chronic obstructive respiratory disease*[tiab] OR lung chronic obstructive disease*[tiab] (111 965)

#2:

"Pulmonary Disease, Chronic Obstructive/rehabilitation"[Mesh] OR "Rehabilitation"[Mesh:NoExp] OR Exercise Therapy[Mesh] OR "Exercise"[Mesh] OR "Bicycling"[Mesh] OR Aerobic training* [tiab] OR aerobic exercise*[tiab] OR exercise training*[tiab] OR rehabilitation[tiab] OR Exercise Therap*[tiab] OR Endurance Training*[tiab] OR Walking[tiab] OR swimming[tiab] OR rowing[tiab] OR endurance exercise*[tiab] OR interval training*[tiab] OR cycling[tiab] OR bicycling[tiab] OR training*[tiab] OR hit[tiab] (941 554)

#3: #1 AND #2 (9 021)

#4: #1 AND #2 Filters: English (7 931)

#5: #1 AND #2 Filters: from 2007 – 3000/12/12 (6 344)

#6: #1 AND #2 Filters: English, from 2007 – 3000/12/12 (5 828)

Total hits: 5 828

Begränsad delning

2. Search strategy for Embase

Date: 27-05-2021

1. *chronic obstructive lung disease/ or *lung emphysema/ or *chronic bronchitis/ or (Chronic Obstructive Pulmonary Disease* or COPD or COAD or Chronic Obstructive Airway Disease* or Chronic Obstructive Lung Disease* or chronic airflow obstruction* or chronic airflow limitation* or Emphysema or Chronic bronchitis or chronic obstructive bronchitis or chronic obstructive bronchopulmonary disease* or chronic obstructive lung disorder* or chronic obstructive pulmonary disorder* or chronic obstructive respiratory disease* or lung chronic obstructive disease*).ti,ab,kw. (170 046)

2. chronic obstructive lung disease/rh or lung emphysema/rh or chronic bronchitis/rh or exercise/ or aerobic exercise/ or endurance training/ or high intensity interval training/ or training/ or rehabilitation/ or pulmonary rehabilitation/ or kinesiotherapy/ or walking/ or swimming/ or rowing/ or cycling/ or (Aerobic training* or aerobic exercise* or exercise training* or rehabilitat* or Exercise Therap* or Endurance Training* or Walking or swimming or rowing or endurance exercise* or interval training* or cycling or bicycling or training* or hit).ti,ab,kw. (1 349 026)

3. 1 and 2 (18 989)

4. limit 3 to english language (16 992)

5. limit 3 to yr="2007 -Current" (14 531)

6. limit 3 to (english language and yr="2007 -Current") (13 740)

7. limit 6 to conference abstract status (6 130)

8. 6 not 7 (7 610)

Total hits: 7 610

Begränsad delning

3. Search strategy for Cinahl

Date: 27-05-2021

S1. (MH "Pulmonary Disease, Chronic Obstructive+") OR (MH "Emphysema") OR TI ("Chronic Obstructive Pulmonary Disease*" OR COPD OR COAD OR "Chronic Obstructive Airway Disease*" OR "Chronic Obstructive Lung Disease*" OR "chronic airflow obstruction*" OR "chronic airflow limitation*" OR Emphysema OR "Chronic bronchitis" OR "chronic obstructive bronchitis" OR "chronic obstructive bronchopulmonary disease*" OR "chronic obstructive lung disorder*" OR "chronic obstructive pulmonary disorder*" OR "chronic obstructive respiratory disease*" OR "lung chronic obstructive disease*") OR AB ("Chronic Obstructive Pulmonary Disease*" OR COPD OR COAD OR "Chronic Obstructive Airway Disease*" OR "Chronic Obstructive Lung Disease*" OR "chronic airflow obstruction*" OR "chronic airflow limitation*" OR Emphysema OR "Chronic bronchitis" OR "chronic obstructive bronchitis" OR "chronic obstructive bronchopulmonary disease*" OR "chronic obstructive lung disorder*" OR "chronic obstructive pulmonary disorder*" OR "chronic obstructive respiratory disease*" OR "lung chronic obstructive disease*") (33 553)

Expanders: Apply equivalent subjects

Search modes: Boolean/Phrase

S2. (MH "Pulmonary Disease, Chronic Obstructive+/RH") OR (MH "Emphysema/RH") OR (MH "Exercise+") OR (MH "Physical Therapy+") OR (MH "Rehabilitation") OR (MH "Rehabilitation, Pulmonary+") OR (MH "Swimming") OR (MH "Rowing") OR (MH "Cycling") OR TI ("Aerobic training*" OR "aerobic exercise*" OR "exercise training*" OR rehabilitat* OR "Exercise Therap*" OR "Endurance Training*" OR Walking OR swimming OR rowing OR "endurance exercise*" OR "interval training*" OR cycling OR bicycling OR training* OR hit) OR AB ("Aerobic training*" OR "aerobic exercise*" OR "exercise training*" OR rehabilitat* OR "Exercise Therap*" OR "Endurance Training*" OR Walking OR swimming OR rowing OR "endurance exercise*" OR "interval training*" OR cycling OR bicycling OR training* OR hit) (511 922)

Expanders: Apply equivalent subjects

Search modes: Boolean/Phrase

S3. S1 AND S2 (4 398)

Expanders: Apply equivalent subjects

Search modes: Boolean/Phrase

S4. S1 AND S2. (4 147)

Expanders: Apply equivalent subjects

Search modes: Boolean/Phrase

Narrow by Language: English

S5. S1 AND S2 (3 344)

Limiters: Published Date: 20070101-20211231

Begränsad delning

Search modes: Boolean/Phrase

Narrow by Language: English

S6. S1 and S2 (3 130)

Limiters: Published Date: 20070101-20211231

Search modes: Boolean/Phrase

Narrow by Language: English

Expanders – Apply equivalent subjects

Total hits: 3 130

4. Cochrane search strategy

Date: 27-05-2021

Expanders: Word variations enabled.

#1: ("Chronic Obstructive Pulmonary Disease" OR COPD OR COAD OR "Chronic Obstructive Airway Disease" OR "Chronic Obstructive Lung Disease" OR "chronic airflow obstruction" OR "chronic airflow limitation" OR Emphysema OR "Chronic bronchitis" OR "chronic obstructive bronchitis" OR "chronic obstructive bronchopulmonary disease" OR "chronic obstructive lung disorder" OR "chronic obstructive pulmonary disorder" OR "chronic obstructive respiratory disease" OR "lung chronic obstructive disease") :ti,ab,kw

#2: ("Aerobic training" OR "aerobic exercise" OR "exercise training" OR rehabilitat* OR "Exercise Therapy" OR "Endurance Training" OR Walking OR swimming OR rowing OR "endurance exercise" OR "interval training" OR cycling OR bicycling OR training OR hit) :ti,ab,kw

#3: #1 AND #2

Cochrane reviews: 38

Trials: 5415

Cochrane protocols: 8, not included

Editorials: 2, not included

Clinical Answers: 2, not included

Total hits: 5453

Begränsad delning

5. PEDro search strategy

Date: 28-05-2021

#1. Chronic respiratory disease (topic) + aerobic training (abstract and title) (86)

- Limit to systematic reviews and clinical trials (84)
- Other chronic respiratory diseases than COPD excluded (33):
- Remaining hits: 51
- Other languages removed (4)

Total records remaining: 47
