# Supplementary material for: Quality of aerobic training description and its relation to intervention efficacy in chronic obstructive pulmonary disease trials: study protocol for a systematic review, meta-analysis and meta-regression
Source: BMJ Open. 2024 May 24;14(5):e084296. doi: 10.1136/bmjopen-2024-084296 (PMC11129019; doi:10.1136/bmjopen-2024-084296)
Supplement: Author's manuscript [file bmjopen-2024-084296.draft_revisions.pdf]

BMJ Open is committed to open peer review. As part of this commitment we make the peer review history of every article we publish publicly available.

When an article is published we post the peer reviewers' comments and the authors' responses online. We also post the versions of the paper that were used during peer review. These are the versions that the peer review comments apply to.

The versions of the paper that follow are the versions that were submitted during the peer review process. They are not the versions of record or the final published versions. They should not be cited or distributed as the published version of this manuscript.

BMJ Open is an open access journal and the full, final, typeset and author-corrected version of record of the manuscript is available on our site with no access controls, subscription charges or pay-per-view fees (http://bmjopen.bmj.com).

If you have any questions on BMJ Open's open peer review process please email info.bmjopen@bmj.com

BMJ Open

Quality of aerobic training description in chronic obstructive pulmonary disease trials: study protocol for a systematic review, meta-analysis, and meta-regression

Journal:	*BMJ Open*
Manuscript ID	bmjopen-2024-084296
Article Type:	Protocol
Date Submitted by the Author:	15-Jan-2024
Complete List of Authors:	Jakobsson, Johan; Umea University, Community Medicine and Rehabilitation, Physiotherapy<br>Stoffels, Anouk ; Radboud Universiteit, Department of Pulmonary Diseases<br>van Hees, Hieronymus; Radboud Universiteit, Department of Pulmonary Diseases<br>De Brandt, Jana; Umeå Universitet, Department of Community Medicine and Rehabilitation, Physiotherapy<br>Nyberg, André; Umeå University, Department of Community Medicine and Rehabilitation, Physiotherapy<br>Klijn, Peter; Merem Pulmonary Rehabilitation Center; Amsterdam University Medical Centres, Department of Pulmonary Medicine
Keywords:	Pulmonary Disease < Lung Diseases, Pulmonary Disease, Chronic Obstructive, SPORTS MEDICINE, REHABILITATION MEDICINE, Physical Therapy Modalities

SCHOLARONE™
Manuscripts

Quality of aerobic training description in Chronic obstructive pulmonary disease trials: study protocol for a systematic review, meta-analysis, and meta-regression

Authors

Johan Jakobsson[1]*#, Anouk A.F. Stoffels#[2], Hieronymus W.H. van Hees[2], Jana De Brandt[1], Andre Nyberg§[1], Peter Klijn§[3, 4]

Affiliations

*Corresponding author, Johan Jakobsson, johan.jakobsson@umu.se, +46 70 271 46 01, Department of Community Medicine and Rehabilitation, Section of Physiotherapy, SE-901 87, Universitetstorget 4, Sweden.

[1]Umeå University, Department of Community Medicine and Rehabilitation, Section of Physiotherapy, Umeå, Sweden.

[2]Department of Pulmonary Diseases, Radboud University Medical Center, Nijmegen, the Netherlands.

[3]Merem Pulmonary Rehabilitation Center, Hilversum, the Netherlands.

[4]Department of Pulmonary Medicine, Amsterdam University Medical Centre, Amsterdam, the Netherlands

#Authors contributed equally as joint first-authors

§Authors contributed equally as senior authors.

Hieronymus W.H. van Hees: Jeroen.vanHees@radboudumc.nl

Anouk A. F. Stoffels: a_stoffels@hotmail.com

Jana De Brandt: jana.de.brandt@umu.se

Andre Nyberg: andre.nyberg@umu.se

Peter Klijn: PKlijn@merem.nl

Word count: 4747 (Excluding abstract, tables, references)

Key words: COPD; Exercise training; pulmonary rehabilitation; pulmonary disease, chronic obstructive; Exercise prescription

For peer review only

For peer review only

For peer review only - http://bmjopen.bmj.com/site/about/guidelines.xhtml

Abstract

Introduction: Chronic Obstructive Pulmonary Disease (COPD) is a major global health concern, characterized by ventilatory constraints, decreased cardiovascular fitness and reduced limb muscle function, profoundly affecting patients' quality of life. Aerobic training plays a crucial role in the treatment of COPD, but the variability in methodologies and incomplete reporting of key components in aerobic training trials limits the assessment of their effectiveness. This systematic review aims to critically evaluate the application of training principles and reporting of key components in aerobic training trials in randomized controlled trials (RCTs) in the COPD literature.

Methods and Analyses: Adhering to the Preferred Reporting Items for Systematic Reviews and Meta-Analyses Protocol guidelines, this review will include RCTs utilizing aerobic training in individuals with COPD. A comprehensive search, following a predefined search strategy will identify studies from MEDLINE, Embase, CINAHL, CENTRAL and PEDro. Studies including people with COPD and any aerobic training intervention will be included. Two reviewers will independently screen abstracts and titles for inclusion. Two reviewers will independently conduct the screening of full-text documents and data extraction. Study quality will be assessed using the Tool for the assESsment of sTudy quality and bias in Exercise, specifically developed for exercise training studies. The certainty of the evidence will be evaluated using the Grading of Recommendations Assessment, Development and Evaluation approach. A systematic synthesis will be provided, with meta-analyses and meta-regression when appropriate.

Ethics and Dissemination: As this review will involve the analysis of published data, ethical approval is not required. The findings of this review will be disseminated through peer-reviewed publications and conference presentations.

PROSPERO registration number: CRD42021247343

For peer review only

For peer review only - http://bmjopen.bmj.com/site/about/guidelines.xhtml

Article Summary

Strengths and limitations of this study

- This protocol is reported according to the Preferred Reporting Items for Systematic Reviews and Meta-Analyses Protocols.
- The protocol includes a comprehensive search strategy and broad inclusion criteria to allow for a comprehensive synthesis of available evidence.
- Exclusion of literature written in languages not known by the research group might leave relevant literature out of the systematic review.
- The quality of included studies will be examined using the Tool for the assESsment of sTudy quality and bias in EXercise specifically developed for trials with exercise interventions.

For peer review only

Background

Chronic obstructive pulmonary disease (COPD) stands as the third leading cause of death[1], with more than 384 million individuals diagnosed globally[2]. The cornerstone of COPD non-pharmacological treatment is exercise training[2 3], which improves cardiorespiratory fitness, muscle function, and overall quality of life. Exercise training also helps to alleviate ventilatory constraints and reduces the burden of symptoms[3]. As for many chronic conditions[4], aerobic training is one recommended part of the management of COPD[3 5]. It is the most applied and frequently studied mode of exercise in COPD[3 6], often investigated using a randomised controlled trial (RCT).

However, the reporting of RCTs is often incomplete, giving rise to the Consolidated Standards of Reporting Trials (CONSORT) statement in 1996[7]. Provided that the field of medicine depends on transparent reporting of RCTs[8], inadequate reporting of trials is troublesome. As a result, the reliability and validity of findings cannot be judged, nor data synthesised for systematic reviews and meta-analyses. Of course, the literature of rehabilitation research and exercise training is not an exception from this[9]. Indeed, exercise training interventions are often poorly described[10], and the field suffers from other concerns regarding methodological rigor and problems with replication[11]. This negatively influences the quality of evidence, as recently concluded by a review investigating methodological rigour and reporting practices in rehabilitation research[9]. In addition, it also contributes to heterogeneity between pulmonary rehabilitation programs and hinders international benchmarking.

Inadequate reporting practices of exercise interventions led to the development of the Consensus on Exercise Reporting Template (CERT) in 2016[10]. As previously stated, it is almost unethical that researchers are still able, almost without any regulation, to design and test exercise interventions that likely have a low potential for being effective[12]. Adequate reporting of an exercise intervention is the first step to help the reader to understand what the researcher did, assess intervention quality and effectiveness, translate research findings into clinical practice, and synthesise data in systematic reviews and meta-analyses for evidence-based guidelines.

Specifically, for exercise training trials, it is important to report all factors influencing the response to training. First, the well-established training principles[13-15] (Table 1) should be applied in designing an exercise program to ensure the most appropriate exercise type and dose to achieve the desired outcome. Secondly, important exercise training variables[10] that contribute to the training stimulus should be reported. These components should cover the FITT-principles (frequency, intensity, time, type) and information on rest, sequence of exercises and program duration[10]. Thirdly, items concerning the external validity, such as eligibility criteria, setting, thorough reporting of participant characteristics, supervision and adherence to interventions must also be described[8 10 16].

We, and others[17 18], have acknowledged a high level of heterogeneity in exercise training protocols and methodological rigor, as well as lacking descriptions of exercise interventions in the COPD literature. This ambiguity complicates the assessment of intervention effectiveness and challenges the replicability of these interventions and their applicability into clinical settings. As such, complete reporting of training interventions is essential. Inadequate reporting of aerobic training interventions has been reviewed previously in other chronic diseases[19-23], while trials in the COPD literature remains to be investigated.

For peer review only - http://bmjopen.bmj.com/site/about/guidelines.xhtml

Aims and objectives

This systematic review aims to investigate if the application of aerobic training principles and the completeness of reporting of key exercise training variables, intervention characteristics and items concerning external validity are present in aerobic training interventions in COPD. The first objective is to investigate the application of adequate exercise training principles and completeness of reporting in aerobic training interventions in the COPD literature. As such, our overarching review question is:

"To which extent does RCTs with an aerobic training intervention in the COPD literature apply and report the principles of exercise training, and how well are the key training variables, intervention and participant characteristics reported in these trials?

Another objective is to meta-analyse the results of aerobic training on exercise-related outcomes, and specifically to investigate if the completeness of reporting is related to efficacy of the interventions provided. As such, the following research question will be addressed:

"Are the efficacy of aerobic training interventions related to the application of exercise training principles and/or a more complete description of key exercise training variables?"

METHODS

The study protocol of this systematic review is reported in accordance with the Preferred Reporting Items for Systematic Reviews and Meta-Analyses Protocols (PRISMA-P) guideline (provided in online supplemental appendix 1). The systematic review was prospectively registered in the International Prospective Register of Systematic Reviews (PROSPERO) on 18th of May 2021 (CRD42021247343). The final review will be conducted and reported according to the PRISMA 2020 guidelines[24].

Eligibility criteria

Studies will be selected according to the criteria below.

Study designs

Randomized controlled trials (RCTs) will be included in this systematic review. Other longitudinal study design, such as controlled non-randomized studies of interventions, cluster trials and prospective comparative cohort studies will be excluded. Studies with a cross-sectional design, retrospective studies, case series, case reports, commentaries and systematic or narrative reviews, will be excluded.

Participants

Any eligible study design with people with COPD being ≥90% of the participants will be considered. Studies with a higher proportion of non-COPD people can be included if the data for the COPD group is, or can be, separated. The COPD diagnosis should be based on a forced expiratory volume in one second (FEV_1) to forced vital capacity (FVC) < 0.7. There will be no eligibility criteria for disease severity, sex, age, body composition or ethnicity. Participants with additional comorbidities will not be excluded, but COPD should be the primary disease of the participants.

Interventions

Any type of aerobic training intervention of the lower limbs which is aerobically demanding is of interest. For example, cycling, walking, swimming, water-based movements, and aerobics. There will be no eligibility criteria for duration of the intervention or number of exercise sessions.

Comparator

Since this study does not intend to compare the effects between two different interventions, there will be no eligibility criteria for comparators. If there is one group with an eligible aerobic training intervention it will be included independent of type of, or the existence of, a control group in this study.

For peer review only - http://bmjopen.bmj.com/site/about/guidelines.xhtml

Outcomes

Studies will be excluded if they do not contain any outcome related to aerobic fitness. One objective of this systematic review is to investigate whether the key training principles are applied, and training components described completely. See “data collection” for which data that will be extracted for this objective.

For the second objective, the following outcomes are eligible:

Primary Outcomes

- 6-minute walking distance (6MWD, meters)
- Incremental shuttle walk test (ISWT, meters)
- Endurance shuttle walk test (ESWT, duration)
- Peak work rate (watts) or VO_2max (ml/min/kg) during cardiopulmonary exercise test (CPET)
- Constant work-rate test (duration)

Secondary outcomes

- Short physical performance battery (SPPB, score)
- Sit-to-stand tests: 5-time sit-to-stand, 30-seconds sit-to-stand or 1-minute sit-to-stand (time or reps)
- Timed-up and go test (TUG; sec)
- Stair climb power test (Watt)
- Steep ramp test (Watt)

Timing

Data from before (“baseline” or “pre”), immediately after the intervention (“post”) and if applicable, additional follow-ups, will be included for intervention studies with more than two time points of outcome assessments.

Setting

There will be no eligibility criteria for type of setting. Examples of different settings could be home-based, hospital-based, inpatient, outpatient, community based or a combination of these.

Timespan

Articles from 1st of January 2007 to the start of this systematic review will be considered. This timespan was chosen since it would include RCTs published after the first joint statement on pulmonary rehabilitation[25] by the American Thoracic Society and the European Respiratory Society in 2006.

Language

Only articles written in English will be included. A list of possibly relevant titles in other languages will be provided.

SEARCH METHODS FOR IDENTIFICATION OF STUDIES

Electronic searches

Studies will be identified from (2007 to start of the systematic review):

- MEDLINE (PubMed interface)
- Embase
- CINAHL

For peer review

For peer review only - http://bmjopen.bmj.com/site/about/guidelines.xhtml

- Cochrane Central Register of Controlled Trials (CENTRAL)
- Pedro

Other resources

The reference list of includes studies will be hand-searched for other relevant studies. Also, other relevant systematic and narrative review studies will be checked for additional studies eligible for inclusion. Conference proceedings will not be included. No grey literature will be searched since we believe it will not add any additional body of literature.

The Cochrane Database of Systematic Reviews and PROSPERO have been searched for existing or ongoing reviews on the topic, with no findings.

Search strategy

To identify relevant studies, a comprehensive search will be performed. The search strategy has been developed by the authors who are researchers in respiratory physiotherapy and clinical exercise physiology, together with a librarian of the Radboud University (Nijmegen, The Netherlands). The original search strategy was developed for PubMed and has been adapted to other databases' syntax and subject headings. Comprehensive searches will be constructed of both index terming (Medical subject headings (MeSH) term), free text terms and synonyms. An English language restriction will be imposed on the search. The search strategy for Pubmed is presented in the online supplemental appendix 2.

Data collection and analysis

Study records

The results from the literature searches will be uploaded to Rayyan[26], a web-tool for collaborative systematic reviews. Screening question for selection will be pilot tested on a few papers.

The search process will be documented, including:

- the name of the database searched.
- the name of the database provider/system used.
- the date when the search was run.
- the years covered by the search.
- the search terms used, hits per search term and number of articles retrieved.

Selection process

Titles and abstracts retrieved from the searches and additional sources will be reviewed independently by two reviewers to identify potentially relevant studies for inclusion based on the eligibility criteria. Studies will be coded as "yes", "no" or "maybe". Studies with "no" from both reviewers will be directly excluded. Studies with two "yes" will be directly included to the next stage. Studies with conflicting decisions will be checked by at least one reviewer for inclusion or exclusion. Then, we will retrieve the full texts of all potentially eligible studies. If needed, authors will be contacted to resolve questions regarding eligibility.

Full text articles of included studies will be independently screened by pairs of reviewers for eligibility. A third reviewer will assess agreement and resolve disagreements on eligibility using a majority rule. Neither of the review authors will be blind to the journal titles, the study authors, or institutions.

For peer review only - http://bmjopen.bmj.com/site/about/guidelines.xhtml

The results of the selection process will be documented reported in sufficient detail to complete a PRISMA flow diagram. Missing papers will be requested from study authors using e-mail. Inter-assessor agreement will be assessed using Kappa statistics.

Data collection process

From each included study two reviewers will independently extract information on study characteristics, participants characteristics, intervention descriptions and outcome data. Data will be entered into a pilot-tested data extraction form developed a priori. All the extracted variables will then be used in the synthesis and quality appraisal of the aerobic training description. A third reviewer will assess agreement between data extractors and resolve disagreements using a majority rule. If needed, a fourth reviewer will be consulted.

The data as outlined below will be extracted. First, it will be reported whether the data is reported ("1") or not reported ("0"), if its insufficiently reported, not clear or not applicable. Secondly, the actual data will be extracted, such as the corresponding data value or a text description. Continuous data will be reported as mean ± SD or median (Q1-Q3). If it is reported otherwise, such as mean ± SEM, this will be noted. The following variables will be extracted:

- Study details: first author name, publication year, title, journal, study design, study objective, inclusion criteria, exclusion criteria and country of execution. If country of execution is not mentioned in the text, it will be derived from where ethical permission have been obtained.

Participants characteristics will be extracted and reported per intervention group. The aerobic training group will always be coded as the intervention group, and any other group will be coded as control. If the study consists of, and is reported in several subgroups, such as per disease severity, data will be reported per subgroup.

Training principles, reporting of participant characteristics, intervention characteristics and training variables will be extracted for our first objective. In addition, outcomes will be extracted for our second objective.

Participant characteristics that will be extracted:

- General
    - Sample size (n)
    - Age (years)
    - Sex distribution (number, ratio)
- Pulmonary function (coded as reported if any of the data below is provided):
    - FEV_1 (L)
    - FEV_1 (% predicted)
    - GOLD stage I, II, III or IV (number or % per group)
    - GOLD stage A, B, C or D (or A, B or E when applicable) (number or % per group)
    - FEV_1/FVC (ratio)
    - RV/TLC (ratio)
    - Dynamic hyperinflation (change in inspiratory capacity, % or L)
    - DLco (ml/min/kPa)
    - DLco (% predicted)
- Body composition (coded as reported if any of the data below is provided):
    - BMI (kg/m^2)
    - FFMI (kg/m^2)
    - FMI (kg/m^2)

For peer review only - http://bmjopen.bmj.com/site/about/guidelines.xhtml

  - Muscle mass (kg)
- Muscle strength (coded as reported if any of the data below is provided):
  - Muscle strength (kg, Nm or as appropriate)
  - Muscle strength (% predicted, with reference to predicted values)
  - Text description of apparatus and type of contraction used in the measurement.
- Exercise capacity: (coded as reported if any of the data below is provided):
  - CPET (VO_2peak mL/min/kg and % predicted, peak watt (W) and % predicted)
  - ISWT (meters, % predicted)
  - 6-MWT (meters, % predicted)
- Exercise tolerance: (coded as reported if any of the data below is provided):
  - CWRT (duration)
  - ESWT (duration)
- Training status: text description regarding previous experience or training status of participants
- Physical activity (CERT item 15): text description (i.e., sedentary, active) or as steps per day, the Physical Activity Level (PAL) or metabolic equivalents (METs).
- Smoking status: average packyears and number of participants per group of never smokers, smokers, former smokers, and non-smokers.
- Comorbidities: frequency per comorbidity, Charlson-comorbidity index or hospital anxiety and depression scale.
- Specific phenotype: text description of specific COPD-phenotypes, for example, frequent exacerbator, alpha-1-antitrypsine deficiency, asthma-COPD-overlap, amongst others.

Aerobic training intervention characteristics that will be extracted:

- Aerobic training objective: rationale regarding the benefits of the exercise program.
- Training device used, including manufacturer and type (CERT item 1). For example, only “cycle ergometer” is insufficient reporting, as type and manufacturer is missing.
- Type of exercise (CERT item 13): For example, walking, cycling, rowing, stair walking.
- Adjuncts (CERT item 10): for example, other exercise modalities, breathing exercises, nutrition interventions, medications, or education.
- Description of control intervention (CERT item 9)
- Place of aerobic training in intervention: standalone, part of pulmonary rehabilitation, as an adjunct or other.
- Supervision (CERT item 2, 4 16a): type of supervision and ratio between supervisors and participants. Only information whether it was supervised or not is insufficient.
- Supervision qualifications: experience, skills, and education of supervisors.
- Setting (CERT item 12): inpatient, outpatient, home-based, community based, hospital based, combination, or other.
- Delivery (CERT item 3): group or individual. If the delivery is in group, the group size should be specified.
- Attendance: number of, or % of, attended sessions.
- Adherence to protocol (CERT item 5, 13, 14, 16b): in addition to the number or percentage of attended sessions, information should be provided on the adherence to the prespecified protocol. Thus, whether performed intensity, duration and total load of the training was performed as planned.
- Safety: information regarding the number and type of adverse events.
- Dropouts: information regarding the number reason for drop-out.

For peer review only - http://bmjopen.bmj.com/site/about/guidelines.xhtml

Training principles that will be extracted are described in Table 1:

Table 1. Exercise training principles

Principle	Criteria used in this review	Examples and elaboration
**Progression:** Over time, adaptations in the physiological component being trained occurs. Progressions refers to the gradual and systematic increase in training stress to maintain tissue overload and thus, stimulate continued training adaptation.	Progression rules should be clearly outlined in the exercise programme, i.e. a detailed prescription of its realization. If terminology such as "as tolerated" is used, it should be clearly stated what is meant and how it is operationalized.	"The duration of the exercise session is increased by five minutes per week." "The exercise intensity is increased by 0-10% every two weeks based on the following progression rules…"
**Overload:** For training adaptations to occur, the exposure of tissues being trained must be exercise above the accustomed-to training stress.	Rationale should be provided if exercise was prescribed adequately in relation to baseline testing or individual exercise capacity. For interventions longer than six weeks, reassessment should be performed during the intervention.	"Exercise intensity was set at 80% of maximal capacity, which was re-tested every fourth week." When overload (intensity) is solely prescribed on baseline testing in interventions longer than six weeks, this principle is not met.
**Specificity:** The closer the training stimulus resembled the characteristic of an activity (mode of exercise, energetic system, muscles trained) the better the training adaptations will be.	The purpose of the exercise training program is specific and targeted to the patients' primary underlying problems known to establish the primary goal of interest. Moreover, outcome testing is performed in activity specific mode.	The intervention must be aligned with the aim. If the aim is to increase cardiovascular fitness, aerobic training should be used and not primarily strength training. This principle is not met if, for example, the efficacy of a walking intervention is evaluated with a cycling test.
**Individuality:** Each individual will respond uniquely to the same training stimulus. Individuality refers to the modification of training to account for an individual's unique capacity and response to training.	A rationale should be provided on how the needs and abilities of any patient are translated in adaptations in individual training programs. Preferably, the training is tailored based on individual training response.	Exercise is prescribed based on workloads corresponding to a percent of peak workload (watt, speed, heart rate) obtained at baseline testing or intra-intervention tests. Also, day-to-day variations in load are based upon individual training response and not a generic rule.
**Initial values:** Individuals with low initials values in the outcome of interest, will show greater improvements compared to those with higher levels.	The program is individually prescribed based on the results of each participant's baseline fitness assessment. Initial values should be obtained with an appropriate exercise test.	Participants with low levels of, e.g., endurance, will be more likely to see a significant change than those with higher endurance levels. This principle is met if, for example, training load (frequency, duration, or intensity) is based upon initial values.

For peer review only - http://bmjopen.bmj.com/site/about/guidelines.xhtml

**Periodization:** Periodization is characterized by a division of training into periods with specific goals and defined by the variation in training variables such as intensity, duration and frequency.	Rationale is provided for the systematic planning of training periods. Description of manipulation of different training components, e.g. intensity, volume, frequency or recovery.	Example of linear periodization description: "For the first 4 weeks, the training target was 3 series of 6 minutes at 50% of pretraining Wmax. Intensity was increased with 5% when subjects completed all 3 series of cycling. When 3 series of 6 minutes cycling were again achieved, loads were increased. After 4 weeks of training, Wmax was reassessed and used for the remaining 6 weeks. Subjects performed five series of 3 minutes using 80% of the new Wmax."
**Reversibility:** Adaptations to exercise training is reversible, meaning that adaptations will return to baseline (detraining) if the training stimulus is removed.	A rationale is provided on how temporarily discontinuation is taken up in the training plan.	Interruptions in the intervention, due to illness, exacerbations or other reasons, must be considered in terms of return-to-training and how the training is recommenced.
The principles of exercise training[14 15 27 28] assessed in this systematic review.		

We have chosen to not include the principle *diminishing returns* in our critical appraisal. We argue that his principle, which states *that the expected degree of improvement in fitness decreases as individuals become more fit, thereby increasing the effort required for further improvements*, is highly difficult to distinctly judge as "yes" or "no", separately from other principles, specifically progression and overload. For example, if both progression and overload is applied, we believe diminishing returns is considered.

For peer review only - http://bmjopen.bmj.com/site/about/guidelines.xhtml

Training variables that will be extracted (extended version of CERT item 13):

- Program duration (weeks)
- Program frequency (sessions/week)
- Exercise intensity (i.e., Watts or km/h)
- Work-phase duration (minutes:seconds)
- Rest periods between series (minutes:seconds): Only applicable for interval training with rest periods, or continuous training were rest periods are allowed.
- Type of rest between series, if applicable (active, passive)
- Intensity of active rest between series, if applicable (i.e., Watts or km/h)
- Rest periods between exercises (minutes:seconds), applicable on rest periods between exercises which are part of the training, including other components than the aerobic training
- Sequence of exercises, if applicable

Outcomes that will be extracted (reported as yes or no and corresponding data):

Primary outcomes

- 6-minute walking distance (6MWD, meters)
- Incremental shuttle walk test (ISWT, meters)
- Endurance shuttle walk test (ESWT, time)
- Peak work rate (watts) or VO_2max (ml/min/kg) during cardiopulmonary exercise test (CPET)
- Constant work-rate test (time, minutes:seconds)

Secondary outcomes

- Short physical performance battery (SPPB, score)
- Sit-to-stand tests: 5-time sit-to-stand, 30-seconds sit-to-stand or 1-minute sit-to-stand (time or reps)
- Timed-up and go test (TUG; sec)
- Stair climb power test (Watt)
- Steep ramp test (Watt)

Outcomes related to cardiorespiratory capacity and exercise tolerance are considered primary given that these are vital objectives in international COPD treatment guidelines [3 5 29].

Risk of bias and study quality assessment

The risk of bias and reporting in eligible studies will be assessed independently by two reviewers. Disagreements will be resolved by consensus or including a third reviewer. No study will be excluded due to high risk of bias, but sensitivity analyses excluding poor-quality studies will be performed. The risk of bias assessment applies to our secondary objective describe in this protocol. Thus, the risk of bias assessment will be presented along with the results regarding the secondary objective.

To assess risk of bias, we will use the TESTEX (Tool for the assESsment of sTudy quality and bias in EXercise) tool. TESTEX[30] is specifically designed for evaluating the risk of bias and reporting in exercise training studies. The TESTEX contains 12 measures: five on study quality and seven on study reporting (online supplemental appendix 3). A total of 15 points can be provided, where higher ratings mean higher quality (lower risk of bias). While no validated cut-off scores are provided for TESTEX, we will categorize the studies according to the average score as "high quality" (≥12 points), "good quality" (7 to 11 points), and "low quality" (≤6 points) as previously described[31].

For peer review only - http://bmjopen.bmj.com/site/about/guidelines.xhtml

Each study will be independently assessed by two reviewers, and discrepancies will be resolved through discussion or consultation with a third reviewer if needed. We will provide a detailed account of the risk of bias for each study, contributing to the overall assessment of the validity and reliability of the evidence. This evaluation will aid in identifying potential sources of bias and in interpreting the findings of the included studies in the context of their methodological strengths and limitations.

Synthesis and quality appraisal of training principles and quality of reporting

Quality appraisal related to our primary objective is performed independently by two reviewers, who are not involved in the included studies. A third reviewer will resolve disagreements and a majority rule will be used.

Given the nature of the data collected for our primary objective, no meta-analysis will be performed. Instead, we will synthesise the data as described below. The synthesis will be guided by relevant items from the PRISMA and synthesis without meta-analysis (SWiM) reporting guidelines. The SWiM[32] guideline is a nine-item checklist to promote transparent reporting of systematic reviews.

The variables listed under participant characteristics, aerobic training intervention characteristics, training principles and training variables will be rated as: “1” (reported), “0” (not reported), “not clear”, “insufficient” or “not applicable (NA)”.

The sum scores of training principles (up to eight points) and training variables (up to nine points) reported per study will be calculated. Training variables will be corrected for the number of NAs, since different numbers of variables might apply to different studies. The count of exercise training principles met within studies will be presented. The number and percentage of studies complying with all training principles and all relevant training variables will be calculated.

We will also critically appraise the included studies using the CERT checklist[10]. Similarly, each of the 19 items (16 items where items no. 7, 14 and 16 are divided into “a” and “b”) in the CERT checklist will be reported as either “1” (reported), “0” (not reported), “not clear”, or “not applicable”. As such, studies will be provided with 0-19 points on CERT-compliance. In addition, the items “objective of aerobic training intervention”, “place of aerobic training intervention”, and “attendance” as listed under intervention characteristics will also be scored (0-3 points). Individual scores per paper will be presented in a supplementary material while a summary score per variable is presented within the main text. The number and percentage of studies complying per CERT item will also be calculated. Tables will be used to present these data descriptively.

Publication details, reporting of participant and intervention characteristics will be narratively synthesised and presented using descriptive statistics to synthesise complete reporting. Summary scores will also be provided for participant characteristics.

Meta-analysis

For our second objective, meta-analyses will be conducted to synthesise the effect of aerobic training interventions on selected outcomes. We are specifically interested in analysing the effects of application of training principles and complete reporting, as described under “Subgroup analysis and investigation of heterogeneity “.

Meta-analyses will be undertaken where meaningful; if the participants, type of intervention and study design are homogenous enough to warrant pooling. We anticipate only continuous data to be reported for the outcomes of interest. Continuous data will be analysed as the mean difference (MD) where outcome data are reported on a uniform scale or standardized mean difference (SMD) when different metrics are used but deemed clinically homogenous. Meta-analyses will be based on change from

For peer review only - http://bmjopen.bmj.com/site/about/guidelines.xhtml

baseline rather than post-intervention values when available. Data extraction, and any needed imputations or will be done according to recommendations in Cochrane Handbook for Systematic Reviews of Interventions Version 6.4. If needed, additional information from the study authors will be sought using e-mail. A random-effects model will be used to allow for random error and inter-study variability.

We will use per-protocol analyses as the effect of interest, where they are reported. If only intention-to-treat data is reported, this will be used for analysis. If both per-protocol and intention-to-treat data are reported, the intention-to-treat data will be used in a sensitivity analysis. Where multiple trial arms are reported in a single study, we will include only relevant ones. If applicable, we will combine intervention arms or reported subgroups as per the recommendations in Cochrane Handbook for Systematic Reviews of Interventions Version 6.4.

Assessment of heterogeneity

Studies included in the review are likely to be diverse methodologically. As such, heterogeneity in the results is to be expected. We will use the I^2 and τ^2 statistics to measure heterogeneity between the studies in each analysis. If we identify substantial heterogeneity ($I^2 \geq 50\%$), we will report it and explore the possible causes by prespecified subgroup analysis. The importance of the I^2 statistic will be interpreted together with the confidence interval and p-value from the $\chi 2$ statistic, as well as the magnitude and direction of effect as described in the in Cochrane Handbook for Systematic Reviews of Interventions Version 6.4.

Assessment of reporting biases

Publication bias threatens the validity of a review's findings. To address this, if we can pool more than 10 studies, we will create and examine the asymmetry of a funnel plot and perform Egger's test to explore possible small study and publication biases. The Funnel plot provides visual aid for detection of publication bias, indicating whether there is a lack of studies reporting negative or insignificant results.

To further investigate and adjust for publication bias, we will employ the trim-and-fill method[33]. This method is designed to identify and correct for asymmetries in the funnel plot that suggest the presence of publication bias. In the event of funnel plot asymmetry, we will use the trim-and-fill method to impute ostensibly missing effect sizes as a sensitivity analysis. Grading of Recommendations Assessment, Development, and Evaluation (GRADE) is used for assessments of the quality of the evidence.

Subgroup analysis and investigation of heterogeneity

We plan to carry out subgroup analyses to investigate the effect of application of training principles and complete reporting on intervention efficacy:

- Applying relevant principles versus not applying relevant principles: This subgroup analysis will be performed per principle.
- Complete or almost complete reporting of training variables (defined as reporting $\geq$85% of relevant variables, e.g., eight out of nine variables) versus non-complete reporting of training variables (defined as reporting <85% relevant variables)
- High versus low compliance with CERT items. No generally accepted cut-offs exist for compliance to the CERT. Therefore, a post-hoc decision will be made on which cut-off to use to enable an adequate comparison with reasonable subgroups.
- Studies published after versus before publication of CERT.

For peer review only - http://bmjopen.bmj.com/site/about/guidelines.xhtml

Other planned subgroup analyses, not related to training principles or complete reporting but important effect modifiers, that will be performed if an adequate number of studies per analysis is available:

- Sex.
- Exercise intensity.
- Duration of intervention (≥15 sessions compared with <15 sessions).
- Severity of COPD (GOLD A and B versus C and D or E).

Additional sub-group analyses may be added if an adequate number of studies per analysis is available. Any sub-analysis that is not pre-specified will be clearly presented as such and presented in a supplementary material.

In addition, a random-effects univariate meta-regression will be performed to examine the association between complete reporting and outcomes variables. As such, mean difference in an outcome is set as the effect estimate and compliance with CERT (number of items), training principles (number of principles) or training variables as explanatory variable.

Sensitivity analysis

We plan to carry out the following sensitivity analyses, removing the following from the primary outcome analyses to examine whether findings are robust to potentially influential decisions:

- Trials judged in the quality assessment to be of low quality.
- Trials with low or no compliance to training principles, defined as applying zero, one or two (out of eight) training principles.
- Trials reporting a low number of training variables (defined as reporting 33% or less of relevant variables).
- Trials with low compliance with the CERT checklist. No generally accepted cut-offs exist for compliance to the CERT. Therefore, a post-hoc decision will be made on which cut-off to use to enable an adequate sensitivity analysis.

We will compare results from a fixed-effect model versus a random-effect model. If we impute SD using correlation coefficients in the synthesis, sensitivity analyses will be performed with different values of the correlation coefficient to determine if the overall results are robust to the imputed correlation coefficients[34]. The sensitivity analyses will be presented in a summary table. Other suitable sensitivity analyses, as identified during the review process, may also be performed, and reported.

Assessment of the certainty of the evidence

We will employ GRADE approach to assess the certainty of the body of evidence related to the included primary and secondary outcomes. The GRADE approach includes assessing evidence across several key dimensions: Risk of bias, imprecision, inconsistency, indirectness, and publication bias. Thus, the quality of the evidence will be graded as 'high', 'moderate', 'low' or 'very low'. We will use the methods and recommendations described in the Cochrane Handbook for Systematic Reviews of Interventions V.6.4 using the GRADEpro software.

Patient and public involvement

Patients and/or the public were not involved in the design, conduct, reporting, or dissemination plans of this research.

For peer review only - http://bmjopen.bmj.com/site/about/guidelines.xhtml

DISCUSSION

This systematic review is designed to critically assess the application of exercise training principles and the completeness of reporting in RCTs with an aerobic training intervention in the COPD literature. This is, to our knowledge, the first systematic review addressing this topic in COPD trials.

The findings of this systematic review are expected to indicate shortcomings in aerobic training interventions and reporting of RCTs in COPD. Based on the findings, we will provide recommendations for future work to enhance the quality of exercise prescription as well as study quality and reporting practices. Identified limitations or strengths in the included studies could inform future guidelines revisions. This, in turn, might lead to more tailored and effective exercise training in pulmonary rehabilitation.

Registration of the systematic review and publication of this protocol promotes transparency in the review process. It also minimises the risk of duplication of similar reviews. We strive for a high methodological standard by adhering to PRISMA-P, and SWiM guidelines. This review is not without limitations. The exclusion of literature written in other languages than English might leave relevant literature out of the systematic review. For this reason, we will report the number of publications that are excluded due to this reason.

ETHICS AND DISSEMINATION

As this study is a systematic review, ethical approval is not required. The systematic review will be submitted for publication in a peer-reviewed journal. We also intend to present the findings at national and international conferences and inform patient organisations.

Amendments

We will conduct the review according to this protocol and justify any deviations in a specific section of the systematic review.

Author Contributions

JJ contributed to the design of the stud, design of the protocol, developed the data synthesis and meta-analysis, and wrote the initial version of the manuscript.

AS contributed to the design of the study and the protocol, developed the search strategy, data extraction and data synthesis process.

HvH contributed to conceptualising the study, the design of the protocol and reviewed the protocol critically for important intellectual content.

JDB contributed to the design of the protocol, developed the data synthesis and meta-analysis, and reviewed the protocol critically for important intellectual content.

AN contributed to conceptualising the study, the design of the protocol, developed the data synthesis and meta-analysis, and reviewed the protocol critically for important intellectual content.

PK conceptualised the study, developed the data synthesis and meta-analysis, as the guarantor for the protocol and reviewed the protocol critically for important intellectual content.

All authors read, approved, and contributed to the final written manuscript.

Funding

This work is undertaken as part of JJ's PhD work, funded by the Swedish Research Council (Vetenskapsrådet), grant number: 2020-01296.

For peer review only

AS was financially supported by Lung Foundation [#5.1.18.232], the Netherlands.

Funders have had no role in the design of this protocol or in the systematic review; nor in the decision to report and publish the results.

Non-financial support has been provided by the library at Radboud University, in developing the search strategy.

Competing interests
None declared.

Patient consent
Not applicable.

For peer review only

For peer review only - http://bmjopen.bmj.com/site/about/guidelines.xhtml

References

1. World Health Organization. The top 10 causes of death World Health Organization; 2020 [cited 2021. Available from: https://www.who.int/news-room/fact-sheets/detail/the-top-10-causes-of-death accessed 2021-09-07 2021.
2. Global Initiative for Chronic Obstructive Lung Disease. Global Strategy for the Diagnosis, Management, and Prevention of Chronic Obstructive Pulmonary Disease 2023 Report 2022 [cited 2023 2022-01-31]. accessed 2023-01-31 2023.
3. Spruit MA, Singh SJ, Garvey C, et al. An official American Thoracic Society/European Respiratory Society statement: key concepts and advances in pulmonary rehabilitation. *Am J Respir Crit Care Med* 2013;188(8):e13-64. doi: 10.1164/rccm.201309-1634ST [published Online First: 2013/10/17]
4. Pedersen BK, Saltin B. Exercise as medicine - evidence for prescribing exercise as therapy in 26 different chronic diseases. *Scand J Med Sci Sports* 2015;25 Suppl 3:1-72. doi: 10.1111/sms.12581 [published Online First: 2015/11/26]
5. McCarthy B, Casey D, Devane D, et al. Pulmonary rehabilitation for chronic obstructive pulmonary disease. *Cochrane Database Syst Rev* 2015(2):Cd003793. doi: 10.1002/14651858.CD003793.pub3 [published Online First: 2015/02/24]
6. Gloeckl R, Marinov B, Pitta F. Practical recommendations for exercise training in patients with COPD. *Eur Respir Rev* 2013;22(128):178-86. doi: 10.1183/09059180.00000513
7. Begg C, Cho M, Eastwood S, et al. Improving the quality of reporting of randomized controlled trials. The CONSORT statement. *JAMA* 1996;276(8):637-9. doi: 10.1001/jama.276.8.637
8. Schulz KF, Altman DG, Moher D. CONSORT 2010 statement: updated guidelines for reporting parallel group randomised trials. *BMJ* 2010;340:c332. doi: 10.1136/bmj.c332 [published Online First: 2010/03/25]
9. Arienti C, Armijo-Olivo S, Minozzi S, et al. Methodological Issues in Rehabilitation Research: A Scoping Review. *Arch Phys Med Rehabil* 2021;102(8):1614-22.e14. doi: 10.1016/j.apmr.2021.04.006 [published Online First: 20210511]
10. Slade SC, Dionne CE, Underwood M, et al. Consensus on Exercise Reporting Template (CERT): Explanation and Elaboration Statement. *Br J Sports Med* 2016;50(23):1428-37. doi: 10.1136/bjsports-2016-096651 [published Online First: 2016/10/07]
11. Mesquida C, Murphy J, Lakens D, et al. Replication concerns in sports and exercise science: a narrative review of selected methodological issues in the field. *Royal Society Open Science* 2022;9(12):220946. doi: 10.1098/rsos.220946
12. Hoogeboom TJ, Kousemaker MC, van Meeteren NL, et al. i-CONTENT tool for assessing therapeutic quality of exercise programs employed in randomised clinical trials. *Br J Sports Med* 2020 doi: 10.1136/bjsports-2019-101630 [published Online First: 2020/11/05]
13. Hoffman J. Physiological Aspects of Sport Training and Performance. Champaign: IL: Human Kinetics 2002.
14. Kasper K. Sports Training Principles. *Curr Sports Med Rep* 2019;18(4)
15. Campbell KL, Neil SE, Winters-Stone KM. Review of exercise studies in breast cancer survivors: attention to principles of exercise training. *Br J Sports Med* 2012;46(13):909-16. doi: 10.1136/bjsm.2010.082719

10.1136/bjsports-2010-082719 [published Online First: 2012/09/26]

16. Thomas JH, Martijn CK, Nico LUvM, et al. i-CONTENT tool for assessing therapeutic quality of exercise programs employed in randomised clinical trials. *Br J Sports Med* 2021;55(20):1153. doi: 10.1136/bjsports-2019-101630
17. Moecke DP, Zhu K, Gill J, et al. A systematic review of exercise studies for individuals hospitalized with an acute exacerbation of chronic obstructive pulmonary disease: Focus on the principles of exercise training. *Chron Respir Dis* 2023;20:14799731231215363. doi: 10.1177/14799731231215363

For peer review only - http://bmjopen.bmj.com/site/about/guidelines.xhtml

18. Westra B, de Wolf S, Bij de Vaate E, et al. Quality of resistance training description in COPD trials: study protocol for a systematic review. *BMJ open* 2019;9(1):e025030. doi: https://dx.doi.org/10.1136/bmjopen-2018-025030
19. Holden S, Rathleff MS, Jensen MB, et al. How can we implement exercise therapy for patellofemoral pain if we don't know what was prescribed? A systematic review. *Br J Sports Med* 2018;52(6):385. doi: 10.1136/bjsports-2017-097547 [published Online First: 20171030]
20. Jo D, Del Bel MJ, McEwen D, et al. A study of the description of exercise programs evaluated in randomized controlled trials involving people with fibromyalgia using different reporting tools, and validity of the tools related to pain relief. *Clin Rehabil* 2019;33(3):557-63. doi: 10.1177/0269215518815931 [published Online First: 2018/12/06]
21. Medysky ME, Bland KA, Neil-Sztramko SE, et al. Attention to the Principles of Exercise Training in Exercise Studies of Persons With Lung Cancer: A Systematic Review. *J Aging Phys Act* 2021:1-11. doi: 10.1123/japa.2020-0269 [published Online First: 2021/05/09]
22. Neil-Sztramko SE, Medysky ME, Campbell KL, et al. Attention to the principles of exercise training in exercise studies on prostate cancer survivors: a systematic review. *BMC Cancer* 2019;19(1):321. doi: 10.1186/s12885-019-5520-9 [published Online First: 2019/04/07]
23. Neil-Sztramko SE, Winters-Stone KM, Bland KA, et al. Updated systematic review of exercise studies in breast cancer survivors: attention to the principles of exercise training. *Br J Sports Med* 2019;53(8):504-12. doi: 10.1136/bjsports-2017-098389 [published Online First: 2017/11/23]
24. Page MJ, McKenzie JE, Bossuyt PM, et al. The PRISMA 2020 statement: an updated guideline for reporting systematic reviews. *BMJ* 2021;372:n71. doi: 10.1136/bmj.n71
25. Nici L, Donner C, Wouters E, et al. American Thoracic Society/European Respiratory Society statement on pulmonary rehabilitation. *Am J Respir Crit Care Med* 2006;173(12):1390-413. doi: 10.1164/rccm.200508-1211ST [published Online First: 2006/06/09]
26. Ouzzani M, Hammady H, Fedorowicz Z, et al. Rayyan—a web and mobile app for systematic reviews. *Systematic Reviews* 2016;5(1):210. doi: 10.1186/s13643-016-0384-4
27. Bland KA, Neil-Sztramko SE, Zadravec K, et al. Attention to principles of exercise training: an updated systematic review of randomized controlled trials in cancers other than breast and prostate. *BMC Cancer* 2021;21(1):1179. doi: 10.1186/s12885-021-08701-y
28. Winters-Stone KM, Neil SE, Campbell KL. Attention to principles of exercise training: a review of exercise studies for survivors of cancers other than breast. *Br J Sports Med* 2014;48(12):987-95. doi: 10.1136/bjsports-2012-091732 [published Online First: 2013/01/08]
29. Nyberg A, Probst V, Vaes AW. Assessments. In: Holland AE, Corso SD, Spruit MA, eds. Pulmonary Rehabilitation: European Respiratory Society 2021:360.
30. Smart NA, Waldron M, Ismail H, et al. Validation of a new tool for the assessment of study quality and reporting in exercise training studies: TESTEX. *Int J Evid Based Healthc* 2015;13(1):9-18. doi: 10.1097/xeb.0000000000000020
31. Stefanakis M, Batalik L, Antoniou V, et al. Safety of home-based cardiac rehabilitation: A systematic review. *Heart Lung* 2022;55:117-26. doi: https://doi.org/10.1016/j.hrtlng.2022.04.016
32. Campbell M, McKenzie JE, Sowden A, et al. Synthesis without meta-analysis (SWiM) in systematic reviews: reporting guideline. *BMJ* 2020;368:l6890. doi: 10.1136/bmj.l6890
33. Duval S, Tweedie R. Trim and Fill: A Simple Funnel-Plot-Based Method of Testing and Adjusting for Publication Bias in Meta-Analysis. *Biometrics* 2000;56(2):455-63.
34. Higgins JPT TJ, Chandler J, Cumpston M, Li T, Page MJ, Welch VA (editors). Cochrane Handbook for Systematic Reviews of Interventions version 6.2 (updated February 2021): Cochrane, 2021, 2021.

For peer review only - http://bmjopen.bmj.com/site/about/guidelines.xhtml

Reporting checklist for protocol of a systematic review and meta analysis.

Based on the PRISMA-P guidelines.

Instructions to authors

Complete this checklist by entering the page numbers from your manuscript where readers will find each of the items listed below.

Your article may not currently address all the items on the checklist. Please modify your text to include the missing information. If you are certain that an item does not apply, please write "n/a" and provide a short explanation.

Upload your completed checklist as an extra file when you submit to a journal.

In your methods section, say that you used the PRISMA-Preporting guidelines, and cite them as:

Moher D, Shamseer L, Clarke M, Ghersi D, Liberati A, Petticrew M, Shekelle P, Stewart LA. Preferred Reporting Items for Systematic Review and Meta-Analysis Protocols (PRISMA-P) 2015 statement. Syst Rev. 2015;4(1):1.

		Reporting Item	Page Number
**Title**			
Identification	#1a	Identify the report as a protocol of a systematic review	1
Update	#1b	If the protocol is for an update of a previous systematic review, identify as such	NA
**Registration**			
	#2	If registered, provide the name of the registry (such as PROSPERO) and registration number	2
**Authors**			
Contact	#3a	Provide name, institutional affiliation, e-mail address of all protocol authors; provide physical mailing address of corresponding author	1
Contribution	#3b	Describe contributions of protocol authors and identify the	17

For peer review only

		guarantor of the review	
**Amendments**			
	#4	If the protocol represents an amendment of a previously completed or published protocol, identify as such and list changes; otherwise, state plan for documenting important protocol amendments	17
**Support**			
Sources	#5a	Indicate sources of financial or other support for the review	17-18
Sponsor	#5b	Provide name for the review funder and / or sponsor	NA
Role of sponsor or funder	#5c	Describe roles of funder(s), sponsor(s), and / or institution(s), if any, in developing the protocol	17-18
**Introduction**			
Rationale	#6	Describe the rationale for the review in the context of what is already known	5
Objectives	#7	Provide an explicit statement of the question(s) the review will address with reference to participants, interventions, comparators, and outcomes (PICO)	6-7
**Methods**			
Eligibility criteria	#8	Specify the study characteristics (such as PICO, study design, setting, time frame) and report characteristics (such as years considered, language, publication status) to be used as criteria for eligibility for the review	6-7
Information sources	#9	Describe all intended information sources (such as electronic databases, contact with study authors, trial registers or other grey literature sources) with planned dates of coverage	7-8
Search strategy	#10	Present draft of search strategy to be used for at least one electronic database, including planned limits, such that it could be repeated	8 and appendix
Study records - data management	#11a	Describe the mechanism(s) that will be used to manage records and data throughout the review	8-9

For peer review only

Study records - selection process	#11b	State the process that will be used for selecting studies (such as two independent reviewers) through each phase of the review (that is, screening, eligibility and inclusion in meta-analysis)	8-9
Study records - data collection process	#11c	Describe planned method of extracting data from reports (such as piloting forms, done independently, in duplicate), any processes for obtaining and confirming data from investigators	8-9
Data items	#12	List and define all variables for which data will be sought (such as PICO items, funding sources), any pre-planned data assumptions and simplifications	9-13
Outcomes and prioritization	#13	List and define all outcomes for which data will be sought, including prioritization of main and additional outcomes, with rationale	13
Risk of bias in individual studies	#14	Describe anticipated methods for assessing risk of bias of individual studies, including whether this will be done at the outcome or study level, or both; state how this information will be used in data synthesis	13 + 16
Data synthesis	#15a	Describe criteria under which study data will be quantitatively synthesised	14-15
Data synthesis	#15b	If data are appropriate for quantitative synthesis, describe planned summary measures, methods of handling data and methods of combining data from studies, including any planned exploration of consistency (such as I2, Kendall's τ)	14-15
Data synthesis	#15c	Describe any proposed additional analyses (such as sensitivity or subgroup analyses, meta-regression)	15-16
Data synthesis	#15d	If quantitative synthesis is not appropriate, describe the type of summary planned	14
Meta-bias(es)	#16	Specify any planned assessment of meta-bias(es) (such as publication bias across studies, selective reporting within studies)	15
Confidence in cumulative evidence	#17	Describe how the strength of the body of evidence will be assessed (such as GRADE)	16

For peer review only

Notes:

- 10: 8 and appendix The PRISMA-P elaboration and explanation paper is distributed under the terms of the Creative Commons Attribution License CC-BY. This checklist was completed on 12. January 2024 using https://www.goodreports.org/, a tool made by the EQUATOR Network in collaboration with Penelope.ai

For peer review only

Online appendix 2.

Draft search strategy for PubMed

Pubmed search 27-05-2021

#1: (111 965)

"Pulmonary Disease, Chronic Obstructive"[Mesh] OR Chronic Obstructive Pulmonary Disease* [tiab] OR COPD [tiab] OR COAD [tiab] OR Chronic Obstructive Airway Disease* [tiab] OR Chronic Obstructive Lung Disease* [tiab] OR chronic airflow obstruction* [tiab] OR chronic airflow limitation*[tiab] OR Emphysema[tiab] OR Chronic bronchitis[tiab] OR chronic obstructive bronchitis[tiab] OR chronic obstructive bronchopulmonary disease*[tiab] OR chronic obstructive lung disorder*[tiab] OR chronic obstructive pulmonary disorder*[tiab] OR chronic obstructive respiratory disease*[tiab] OR lung chronic obstructive disease*[tiab]

#2: (941 554)

"Pulmonary Disease, Chronic Obstructive/rehabilitation"[Mesh] OR "Rehabilitation"[Mesh:NoExp] OR Exercise Therapy[Mesh] OR "Exercise"[Mesh] OR "Bicycling"[Mesh] OR Aerobic training* [tiab] OR aerobic exercise*[tiab] OR exercise training*[tiab] OR rehabilitation[tiab] OR Exercise Therap*[tiab] OR Endurance Training*[tiab] OR Walking[tiab] OR swimming[tiab] OR rowing[tiab] OR endurance exercise*[tiab] OR interval training*[tiab] OR cycling[tiab] OR bicycling[tiab] OR training*[tiab] OR hit[tiab]

#3: #1 AND #2 (9 021)

#4: #1 AND #2 Filters: English (7 931)

#5: #1 AND #2 Filters: from 2007 – 3000/12/12 (6 344)

#6: #1 AND #2 Filters: English, from 2007 – 3000/12/12 (5 828)

For peer review only

BMJ Open

Quality of aerobic training description and its relation to intervention efficacy in Chronic obstructive pulmonary disease trials: study protocol for a systematic review, meta-analysis, and meta-regression

Journal:	*BMJ Open*
Manuscript ID	bmjopen-2024-084296.R1
Article Type:	Protocol
Date Submitted by the Author:	03-Apr-2024
Complete List of Authors:	Jakobsson, Johan; Umea University, Community Medicine and Rehabilitation, Physiotherapy<br>Stoffels, Anouk ; Radboud Universiteit, Department of Pulmonary Diseases<br>van Hees, Hieronymus; Radboud Universiteit, Department of Pulmonary Diseases<br>De Brandt, Jana; Umeå Universitet, Department of Community Medicine and Rehabilitation, Physiotherapy<br>Nyberg, André; Umeå University, Department of Community Medicine and Rehabilitation, Physiotherapy<br>Klijn, Peter; Merem Pulmonary Rehabilitation Center; Amsterdam University Medical Centres, Department of Pulmonary Medicine
**Primary Subject Heading**:	Respiratory medicine
Secondary Subject Heading:	Sports and exercise medicine, Rehabilitation medicine, Public health
Keywords:	Pulmonary Disease < Lung Diseases, Pulmonary Disease, Chronic Obstructive, SPORTS MEDICINE, REHABILITATION MEDICINE, Physical Therapy Modalities

SCHOLARONE™
Manuscripts

endixQuality of aerobic training description and its relation to intervention efficacy in Chronic obstructive pulmonary disease trials: study protocol for a systematic review, meta-analysis, and meta-regression

Authors

Johan Jakobsson[1]*#, Anouk A.F. Stoffels#[2], Hieronymus W.H. van Hees[2], Jana De Brandt[1], Andre Nyberg§[1], Peter Klijn§[3, 4]

Affiliations

*Corresponding author, Johan Jakobsson, johan.jakobsson@umu.se, +46 70 271 46 01, Department of Community Medicine and Rehabilitation, Section of Physiotherapy, SE-901 87, Universitetstorget 4, Sweden.

[1]Umeå University, Department of Community Medicine and Rehabilitation, Section of Physiotherapy, Umeå, Sweden.

[2]Department of Pulmonary Diseases, Radboud University Medical Center, Nijmegen, the Netherlands.

[3]Merem Pulmonary Rehabilitation Center, Hilversum, the Netherlands.

[4]Department of Pulmonary Medicine, Amsterdam University Medical Centre, Amsterdam, the Netherlands

#Authors contributed equally as joint first authors

§Authors contributed equally as senior authors.

Hieronymus W.H. van Hees: Jeroen.vanHees@radboudumc.nl

Anouk A. F. Stoffels: a_stoffels@hotmail.com

Jana De Brandt: jana.de.brandt@umu.se

Andre Nyberg: andre.nyberg@umu.se

Peter Klijn: PKlijn@merem.nl

Word count: 4964 (Excluding abstract, tables, references)

Key words: COPD; Exercise training; pulmonary rehabilitation; pulmonary disease, chronic obstructive; Exercise prescription

For peer review only

For peer review only

For peer review only - http://bmjopen.bmj.com/site/about/guidelines.xhtml

Abstract

Introduction: Chronic Obstructive Pulmonary Disease (COPD) is a major global health concern, characterized by ventilatory constraints, decreased cardiovascular fitness and reduced limb muscle function, profoundly affecting patients' quality of life. Aerobic training plays a crucial role in the treatment of COPD, but the variability in methodologies and incomplete reporting of key components in aerobic training trials limits the assessment of their effectiveness. This systematic review aims to critically evaluate the application of training principles and reporting of key components in aerobic training trials in randomized controlled trials (RCTs) in the COPD literature.

Methods and Analysis: The protocol adheres to the Preferred Reporting Items for Systematic Reviews and Meta Analyses Protocol guidelines. The review will include RCTs utilizing aerobic training in individuals with COPD. A comprehensive search, following a predefined search strategy will identify studies published from 2007 to 2021 in English from MEDLINE, Embase, CINAHL, CENTRAL and PEDro. Studies including people with COPD and any aerobic training intervention will be included. Two reviewers will independently screen abstracts and titles for inclusion. Two reviewers will independently conduct the screening of full-text documents and data extraction. Study quality will be assessed using the Tool for the assESsment of sTudy quality and bias in Exercise, specifically developed for exercise training studies. The certainty of the evidence will be evaluated using the Grading of Recommendations Assessment, Development and Evaluation approach. A systematic synthesis will be provided, with meta-analyses and meta-regression when appropriate.

Ethics and Dissemination: As this review will involve the analysis of published data, ethical approval is not required. The findings of this review will be disseminated through peer-reviewed publications and conference presentations.

PROSPERO registration number: CRD42021247343

For peer review only

For peer review only - http://bmjopen.bmj.com/site/about/guidelines.xhtml

Article Summary

Strengths and limitations of this study

- This protocol is reported according to the Preferred Reporting Items for Systematic Reviews and Meta-Analyses Protocols.
- The protocol includes a comprehensive search strategy and broad inclusion criteria to allow for a comprehensive synthesis of available evidence.
- We will examine the quality of included studies using the Tool for the assESsment of sTudy quality and bias in EXercise specifically developed for trials with exercise interventions.
- Exclusion of literature written in languages not known by the research group might leave relevant literature out of the systematic review.

For peer review only

For peer review only - http://bmjopen.bmj.com/site/about/guidelines.xhtml

INTRODUCTION

Chronic obstructive pulmonary disease (COPD) stands as the third leading cause of death[1], with more than 384 million individuals diagnosed globally[2]. In the management of COPD, the beneficial impacts of pulmonary rehabilitation, incorporating aerobic exercise training as a key element, are well-documented[2-5]. These improvements typically manifest in increased cardiorespiratory fitness[6], better overall quality of life and it reduces the burden of symptoms[3 7-9]. In fact, among different exercises modalities within PR, aerobic training is the most frequently applied and investigated mode of exercise in COPD[3 10], often investigated using a randomised controlled trial (RCT).

However, the reporting of RCTs is often incomplete, giving rise to the Consolidated Standards of Reporting Trials (CONSORT) statement in 1996[11]. Provided that the field of medicine depends on transparent reporting of RCTs[12], inadequate reporting of trials is troublesome. As a result, the reliability and validity of findings cannot be judged, nor data synthesised for systematic reviews and meta-analyses[13]. Of course, the literature of rehabilitation research and exercise training is not an exception from this[14]. Indeed, exercise training interventions are often poorly described[15], and the field suffers from other concerns regarding methodological rigor and problems with replication[16]. This negatively influences the quality of evidence, as recently concluded by a review investigating methodological rigour and reporting practices in rehabilitation research[14]. In addition, it also contributes to heterogeneity between pulmonary rehabilitation programs and hinders international benchmarking.

Inadequate reporting practices of exercise interventions led to the development of the Consensus on Exercise Reporting Template (CERT) in 2016[15]. As previously stated, it is almost unethical that researchers are still able, almost without any regulation, to design and test exercise interventions that likely have a low potential for being effective[17]. Adequate reporting of research, including exercise interventions, is the first step to help the reader to understand what the researcher did, assess intervention quality and effectiveness, translate research findings into clinical practice, and synthesise data in systematic reviews and meta-analyses for evidence-based guidelines. Although the efficacy of aerobic training interventions in COPD is evident, we, and others[18 19], have acknowledged a high level of heterogeneity in exercise training protocols and methodological rigor, as well as lacking descriptions of exercise interventions in the COPD literature. This ambiguity complicates the assessment of intervention effectiveness and challenges the replicability of these interventions and their applicability into clinical settings. As such, complete reporting of training interventions is essential. Inadequate reporting of aerobic training interventions has been reviewed previously in other chronic diseases[20-24], while trials in the COPD literature remains to be investigated.

Specifically, for exercise training trials, it is important to report all factors influencing the response to training. First, the well-established training principles[25-27] (Table 1) should be applied in designing an exercise program to ensure the most appropriate exercise type and dose to achieve the desired outcome. Secondly, important exercise training variables[15] that contribute to the training stimulus should be reported. These components should cover the FITT-principles (frequency, intensity, time, type) and information on rest, sequence of exercises and program duration[15]. Evidence on the importance of incorporating the FITT-principles within aerobic training studies are starting to emerge. Recent data suggests that FITT-principles such as frequency and time seem to be associated with an increase in peak oxygen uptake following aerobic training in individuals with COPD[6]. Thirdly, items concerning the external validity, such as eligibility criteria, setting, thorough reporting of participant characteristics, supervision and adherence to interventions must also be described[12 15 28 29]. Still, whether the efficacy of aerobic training interventions among people with COPD is related to the

For peer review only - http://bmjopen.bmj.com/site/about/guidelines.xhtml

application of exercise training principles and/or a more complete description of key exercise training variables remains to be fully determined.

Aims and objectives

We aim to investigate the application of exercise training principles and completeness of reporting in aerobic training interventions in the COPD literature. We also aim to investigate if efficacy of the interventions provided is related to the completeness of reporting and application of training principles. Our specific research questions are:

"To what extent does RCTs with an aerobic training intervention in the COPD literature apply and report the principles of exercise training, and how well are the key training variables, intervention and participant characteristics reported in these trials?

"Is the efficacy of aerobic training interventions related to the application of exercise training principles and/or a more complete description of key exercise training variables?"

METHODS AND ANALYSIS

The study protocol of this systematic review is reported in accordance with the Preferred Reporting Items for Systematic Reviews and Meta-Analyses Protocols (PRISMA-P) guideline (provided in online supplemental appendix 1). The systematic review was prospectively registered in the International Prospective Register of Systematic Reviews (PROSPERO) on 18th of May 2021 (CRD42021247343). The final review will be reported according to the PRISMA 2020 guidelines[30].

Eligibility criteria

We will select studies according to the criteria below.

Study designs

Only RCTs will be included in this systematic review. Other longitudinal study design, such as controlled non-randomized studies of interventions, cluster trials and prospective comparative cohort studies will be excluded. Studies with a cross-sectional design, retrospective studies, case series, case reports, commentaries and systematic or narrative reviews, will be excluded.

Participants

Any eligible study design with people with COPD being ≥ 90% of the participants will be considered. Studies with a higher proportion of non-COPD people can be included if the data for the COPD group is, or can be, separated. The COPD diagnosis should be based on a forced expiratory volume in one second (FEV_1) to forced vital capacity (FVC) < 0.7. There will be no eligibility criteria for disease severity, sex, age, body composition or ethnicity. Participants with additional comorbidities will not be excluded, but COPD should be the primary disease of the participants.

Interventions

Any type of aerobic training intervention of the lower limbs which is aerobically demanding is of interest. For example, cycling, walking, swimming, water-based movements, and aerobics. There will be no eligibility criteria for duration of the intervention or number of exercise sessions.

Comparator

Since this study does not intend to compare the effects between two different interventions, there will be no eligibility criteria for comparators. If there is one group with an eligible aerobic training intervention it will be included independent of type of, or the existence of, a control group in this study.

Outcomes

For peer review only - http://bmjopen.bmj.com/site/about/guidelines.xhtml

During the screening phase, studies will not be excluded based on outcomes. One objective of this systematic review is to investigate whether the key training principles are applied, and whether training components are described completely. See "data collection" for which data that will be extracted for this objective.

For the second objective, the following outcomes are eligible:

Primary Outcomes

- 6-minute walking distance (6MWD, meters)
- Incremental shuttle walk test (ISWT, meters)
- Endurance shuttle walk test (ESWT, duration)
- Peak work rate (watts) or VO_2max (ml/min/kg) during cardiopulmonary exercise test (CPET)
- Constant work-rate test (duration)

Secondary outcomes

- Short physical performance battery (SPPB, score)
- Sit-to-stand tests: 5-time sit-to-stand, 30-seconds sit-to-stand or 1-minute sit-to-stand (time or reps)
- Timed-up and go test (TUG; sec)
- Stair climb power test (Watt)
- Steep ramp test (Watt)

Timing

Data from before ("baseline" or "pre"), immediately after the intervention ("post") and if applicable, additional follow-ups, will be included for intervention studies with more than two time points of outcome assessments.

Setting

There will be no eligibility criteria for type of setting. Examples of different settings could be home-based, hospital-based, inpatient, outpatient, community based or a combination of these.

Timespan

Articles from 1st of January 2007 to 27th of May 2021 will be considered. This timespan was chosen since it would include RCTs published after the first joint statement on pulmonary rehabilitation[31] by the American Thoracic Society and the European Respiratory Society in 2006.

Language

Only articles written in English will be included. A list of possibly relevant titles in other languages will be provided.

SEARCH METHODS FOR IDENTIFICATION OF STUDIES

Electronic searches

Studies will be identified from :

- MEDLINE (PubMed interface)
- Embase
- CINAHL
- Cochrane Central Register of Controlled Trials (CENTRAL)
- PEDro

For peer review only - http://bmjopen.bmj.com/site/about/guidelines.xhtml

Other resources

The reference list of includes studies will be hand-searched for other relevant studies. Also, other relevant systematic and narrative review studies will be checked for additional studies eligible for inclusion. Conference proceedings will not be included. No grey literature will be searched since we believe it will not add any additional body of literature.

The Cochrane Database of Systematic Reviews and PROSPERO have been searched for existing or ongoing reviews on the topic, with no findings.

Search strategy

To identify relevant studies, the comprehensive search was performed on 27th May 2021. The search strategy has been developed by the authors who are researchers in respiratory physiotherapy and clinical exercise physiology, together with a librarian of the Radboud University (Nijmegen, The Netherlands). The original search strategy was developed for PubMed and has been adapted to other databases' syntax and subject headings. Comprehensive searches was constructed of both index terming (Medical subject headings (MeSH) term), free text terms and synonyms. An English language restriction was imposed on the search. The search strategy for the electronic searches is presented in the online supplemental appendix 2.

Data collection and analysis

Study records

The results from the literature searches will be uploaded to Rayyan[32], a web-tool for collaborative systematic reviews. Screening question for selection will be pilot tested on a few papers.

The search process will be documented, including:

- the name of the database searched.
- the name of the database provider/system used.
- the date when the search was run.
- the years covered by the search.
- the search terms used, hits per search term and number of articles retrieved.

Selection process

Titles and abstracts retrieved from the searches and additional sources will be reviewed independently by two reviewers to identify potentially relevant studies for inclusion based on the eligibility criteria. Studies will be coded as "yes", "no" or "maybe". Studies with "no" from both reviewers will be directly excluded. Studies with two "yes" will be directly included to the next stage. Studies with conflicting decisions will be checked by at least one reviewer for inclusion or exclusion. Then, we will retrieve the full texts of all potentially eligible studies. If needed, authors will be contacted to resolve questions regarding eligibility.

Full text articles of included studies will be independently screened by pairs of reviewers for eligibility. A third reviewer will assess agreement and resolve disagreements on eligibility using a majority rule. Neither of the review authors will be blind to the journal titles, the study authors, or institutions.

The results of the selection process will be documented reported in sufficient detail to complete a PRISMA flow diagram. Missing papers will be requested from study authors using e-mail. Inter-assessor agreement will be assessed using Kappa statistics.

Data collection process

For peer review only - http://bmjopen.bmj.com/site/about/guidelines.xhtml

From each included study two reviewers will independently extract information on study characteristics, participants characteristics, intervention descriptions and outcome data. Data will be entered into a pilot-tested data extraction form developed a priori. All the extracted variables will then be used in the synthesis and quality appraisal of the aerobic training description. A third reviewer will assess agreement between data extractors and resolve disagreements using a majority rule. If needed, a fourth reviewer will be consulted.

The data as outlined below will be extracted. First, it will be reported whether the data is reported ("1") or not reported ("0"), if its insufficiently reported, not clear or not applicable. Secondly, the actual data will be extracted, such as the corresponding data value or a text description. Continuous data will be reported as mean ± SD or median (Q1-Q3). If it is reported otherwise, such as mean ± SEM, this will be noted. The following variables will be extracted:

- Study details: first author name, publication year, title, journal, study design, study objective, inclusion criteria, exclusion criteria and country of execution. If country of execution is not mentioned in the text, it will be derived from where ethical permission have been obtained.

Participants characteristics will be extracted and reported per intervention group. The aerobic training group will always be coded as the intervention group, and any other group will be coded as control. If the study consists of, and is reported in several subgroups, such as per disease severity, data will be reported per subgroup.

Training principles, reporting of participant characteristics, intervention characteristics and training variables will be extracted for our first objective. In addition, outcomes will be extracted for our second objective.

Participant characteristics that will be extracted:

- General
    - Sample size (n)
    - Age (years)
    - Sex distribution (number, ratio)
- Pulmonary function (coded as reported if any of the data below is provided):
    - FEV_1 (L)
    - FEV_1 (% predicted)
    - GOLD stage I, II, III or IV (number or % per group)
    - GOLD stage A, B, C or D (or A, B or E when applicable) (number or % per group)
    - FEV_1/FVC (ratio)
    - RV/TLC (ratio)
    - Dynamic hyperinflation (change in inspiratory capacity, % or L)
    - DLco (ml/min/kPa)
    - DLco (% predicted)
- Body composition (coded as reported if any of the data below is provided):
    - BMI (kg/m^2)
    - FFMI (kg/m^2)
    - FMI (kg/m^2)
    - Muscle mass (kg)
- Muscle strength (coded as reported if any of the data below is provided):
    - Muscle strength (kg, Nm or as appropriate)
    - Muscle strength (% predicted, with reference to predicted values)

For peer review only

For peer review only - http://bmjopen.bmj.com/site/about/guidelines.xhtml

- Text description of apparatus and type of contraction used in the measurement.
- Exercise capacity: (coded as reported if any of the data below is provided):
  - CPET (VO_2peak mL/min/kg and % predicted, peak watt (W) and % predicted)
  - ISWT (meters, % predicted)
  - 6-MWT (meters, % predicted)
- Exercise tolerance: (coded as reported if any of the data below is provided):
  - CWRT (duration)
  - ESWT (duration)
- Training status: text description regarding previous experience or training status of participants
- Physical activity (CERT item 15): text description (i.e., sedentary, active) or as steps per day, the Physical Activity Level (PAL) or metabolic equivalents (METs).
- Smoking status: average packyears and number of participants per group of never smokers, smokers, former smokers, and non-smokers.
- Comorbidities: frequency per comorbidity, Charlson-comorbidity index or hospital anxiety and depression scale.
- Specific phenotype: text description of specific COPD-phenotypes, for example, frequent exacerbator, alpha-1-antitrypsine deficiency, asthma-COPD-overlap, amongst others.

Aerobic training intervention characteristics that will be extracted:

- Aerobic training objective: rationale regarding the benefits of the exercise program.
- Training device used, including manufacturer and type (CERT item 1). For example, only “cycle ergometer” is insufficient reporting, as type and manufacturer is missing.
- Type of exercise (CERT item 13): For example, walking, cycling, rowing, stair walking.
- Adjuncts (CERT item 10): for example, other exercise modalities, breathing exercises, nutrition interventions, medications, or education.
- Description of control intervention (CERT item 9)
- Place of aerobic training in intervention: standalone, part of pulmonary rehabilitation, as an adjunct or other.
- Supervision (CERT item 2, 4 16a): type of supervision and ratio between supervisors and participants. Only information whether it was supervised or not is insufficient.
- Supervision qualifications: experience, skills, and education of supervisors.
- Setting (CERT item 12): inpatient, outpatient, home-based, community based, hospital based, combination, or other.
- Delivery (CERT item 3): group or individual. If the delivery is in group, the group size should be specified.
- Attendance: number of, or % of, attended sessions.
- Adherence to protocol (CERT item 5, 13, 14, 16b): in addition to the number or percentage of attended sessions, information should be provided on the adherence to the prespecified protocol. Thus, whether performed intensity, duration and total load of the training was performed as planned.
- Safety: information regarding the number and type of adverse events.
- Dropouts: information regarding the number reason for drop-out.

For peer review only

For peer review only - http://bmjopen.bmj.com/site/about/guidelines.xhtml

Training principles that will be extracted are described in Table 1:

Table 1. Exercise training principles

Principle	Criteria used in this review	Examples and elaboration
**Progression:** Over time, adaptations in the physiological component being trained occurs. Progressions refers to the gradual and systematic increase in training stress to maintain tissue overload and thus, stimulate continued training adaptation.	Progression rules should be clearly outlined in the exercise programme, i.e. a detailed prescription of its realization. If terminology such as "as tolerated" is used, it should be clearly stated what is meant and how it is operationalized.	"The duration of the exercise session is increased by five minutes per week."<br><br>"The exercise intensity is increased by 0-10% every two weeks based on the following progression rules..."
**Overload:** For training adaptations to occur, the exposure of tissues being trained must be exercise above the accustomed-to training stress.	Rationale should be provided if exercise was prescribed adequately in relation to baseline testing or individual exercise capacity. For interventions longer than six weeks, reassessment should be performed during the intervention.	"Exercise intensity was set at 80% of maximal capacity, which was re-tested every fourth week."<br><br>When overload (intensity) is solely prescribed on baseline testing in interventions longer than six weeks, this principle is not met.
**Specificity:** The closer the training stimulus resembled the characteristic of an activity (mode of exercise, energetic system, muscles trained) the better the training adaptations will be.	The purpose of the exercise training program is specific and targeted to the patients' primary underlying problems known to establish the primary goal of interest. Moreover, outcome testing is performed in activity specific mode.	The intervention must be aligned with the aim. If the aim is to increase cardiovascular fitness, aerobic training should be used and not primarily strength training. This principle is not met if, for example, the efficacy of a walking intervention is evaluated with a cycling test.
**Individuality:** Each individual will respond uniquely to the same training stimulus. Individuality refers to the modification of training to account for an individual's unique capacity and response to training.	A rationale should be provided on how the needs and abilities of any patient are translated in adaptations in individual training programs. Preferably, the training is tailored based on individual training response.	Exercise is prescribed based on workloads corresponding to a percent of peak workload (watt, speed, heart rate) obtained at baseline testing or intra-intervention tests. Also, day-to-day variations in load are based upon individual training response and not a generic rule.
**Initial values:** Individuals with low initials values in the outcome of interest, will show greater improvements compared to those with higher levels.	The program is individually prescribed based on the results of each participant's baseline fitness assessment. Initial values should be obtained with an appropriate exercise test.	Participants with low levels of, e.g., endurance, will be more likely to see a significant change than those with higher endurance levels. This principle is met if, for example, training load (frequency, duration, or intensity) is based upon initial values.

For peer review only - http://bmjopen.bmj.com/site/about/guidelines.xhtml

**Periodization:** Periodization is characterized by a division of training into periods with specific goals and defined by the variation in training variables such as intensity, duration and frequency.	Rationale is provided for the systematic planning of training periods. Description of manipulation of different training components, e.g. intensity, volume, frequency or recovery.	Example of linear periodization description: "For the first 4 weeks, the training target was 3 series of 6 minutes at 50% of pretraining maximum work rate. Intensity was increased with 5% when subjects completed all 3 series of cycling. When 3 series of 6 minutes cycling were again achieved, loads were increased. After 4 weeks of training, Wmax was reassessed and used for the remaining 6 weeks. Subjects performed five series of 3 minutes using 80% of the new Wmax."
**Reversibility:** Adaptations to exercise training is reversible, meaning that adaptations will return to baseline (detraining) if the training stimulus is removed.	A rationale is provided on how temporarily discontinuation is taken up in the training plan.	Interruptions in the intervention, due to illness, exacerbations or other reasons, must be considered in terms of return-to-training and how the training is recommenced.
The principles of exercise training[26 27 33 34] assessed in this systematic review.		

We have chosen to not include the principle *diminishing returns* in our critical appraisal. We argue that his principle, which states *that the expected degree of improvement in fitness decreases as individuals become more fit, thereby increasing the effort required for further improvements*, is highly difficult to distinctly judge as "yes" or "no", separately from other principles, specifically progression and overload. For example, if both progression and overload is applied, we believe diminishing returns is considered.

For peer review only - http://bmjopen.bmj.com/site/about/guidelines.xhtml

Training variables that will be extracted (extended version of CERT item 13):

- Program duration (weeks)
- Program frequency (sessions/week)
- Exercise intensity (i.e., Watts or km/h)
- Work-phase duration (minutes: seconds)
- Rest periods between series (minutes: seconds): Only applicable for interval training with rest periods, or continuous training were rest periods are allowed.
- Type of rest between series, if applicable (active, passive)
- Intensity of active rest between series, if applicable (i.e., Watts or km/h)
- Rest periods between exercises (minutes: seconds), applicable on rest periods between exercises which are part of the training, including other components than the aerobic training
- Sequence of exercises, if applicable

Outcomes that will be extracted (reported as yes or no and corresponding data):

Primary outcomes

- 6-minute walking distance (6MWD, meters)
- Incremental shuttle walk test (ISWT, meters)
- Endurance shuttle walk test (ESWT, time)
- Peak work rate (watts) or VO_2max (ml/min/kg) during cardiopulmonary exercise test (CPET)
- Constant work-rate test (time, minutes: seconds)

Secondary outcomes

- Short physical performance battery (SPPB, score)
- Sit-to-stand tests: 5-time sit-to-stand, 30-seconds sit-to-stand or 1-minute sit-to-stand (time or reps)
- Timed-up and go test (TUG; sec)
- Stair climb power test (Watt)
- Steep ramp test (Watt)

We consider outcomes related to cardiorespiratory capacity and exercise tolerance primary given that these are vital objectives in international COPD treatment guidelines [3 29 35].

Risk of bias and study quality assessment

The risk of bias and reporting in eligible studies will be assessed independently by two reviewers. Disagreements will be resolved by consensus or including a third reviewer. No study will be excluded due to high risk of bias, but sensitivity analyses excluding poor-quality studies will be performed. The risk of bias assessment applies to our secondary objective describe in this protocol. Thus, the risk of bias assessment will be presented along with the results regarding the secondary objective.

To assess risk of bias, we will use the TESTEX (Tool for the assESsment of sTudy quality and bias in EXercise) tool. TESTEX[36] is specifically designed for evaluating the risk of bias and reporting in exercise training studies. The TESTEX contains 12 measures: five on study quality and seven on study reporting. A total of 15 points can be provided, where higher ratings mean higher quality (lower risk of bias). While no validated cut-off scores are provided for TESTEX, we will categorize the studies according to the average score as "high quality" (≥12 points), "good quality" (7 to 11 points), and "low quality" (≤6 points) as previously described[37].

For peer review only

For peer review only - http://bmjopen.bmj.com/site/about/guidelines.xhtml

Each study will be independently assessed by two reviewers, and discrepancies will be resolved through discussion or consultation with a third reviewer if needed. We will provide a detailed account of the risk of bias for each study, contributing to the overall assessment of the validity and reliability of the evidence. This evaluation will aid in identifying potential sources of bias and in interpreting the findings of the included studies in the context of their methodological strengths and limitations.

Synthesis and quality appraisal of training principles and quality of reporting

Quality appraisal related to our primary objective is performed independently by two reviewers, who are not involved in the included studies. A third reviewer will resolve disagreements and a majority rule will be used.

Given the nature of the data collected for our primary objective, no meta-analysis will be performed. Instead, we will synthesise the data as described below. The synthesis will be guided by relevant items from the PRISMA and synthesis without meta-analysis (SWiM) reporting guidelines. The SWiM[38] guideline is a nine-item checklist to promote transparent reporting of systematic reviews.

The variables listed under participant characteristics, aerobic training intervention characteristics, training principles and training variables will be rated as: “1” (reported), “0” (not reported), “not clear”, “insufficient” or “not applicable (NA)”.

The sum scores of training principles (up to eight points) and training variables (up to nine points) reported per study will be calculated. Training variables will be corrected for the number of NAs, since different numbers of variables might apply to different studies. The count of exercise training principles met within studies will be presented. The number and percentage of studies complying with all training principles and all relevant training variables will be calculated.

We will also critically appraise the included studies using the CERT checklist[15]. Similarly, each of the 19 items (16 items where items no. 7, 14 and 16 are divided into “a” and “b”) in the CERT checklist will be reported as either “1” (reported), “0” (not reported), “not clear”, or “not applicable”. As such, studies will be provided with 0-19 points on CERT-compliance. In addition, the items “objective of aerobic training intervention”, “place of aerobic training intervention”, and “attendance” as listed under intervention characteristics will also be scored (0-3 points). Individual scores per paper will be presented in a supplementary material while a summary score per variable is presented within the main text. The number and percentage of studies complying per CERT item will also be calculated. Tables will be used to present these data descriptively.

Publication details, reporting of participant and intervention characteristics will be narratively synthesised and presented using descriptive statistics to synthesise complete reporting. Summary scores will also be provided for participant characteristics.

Meta-analysis

For our second objective, meta-analyses will be conducted to synthesise the efficacy of aerobic training interventions on selected outcomes. We are specifically interested in analysing the effects of application of training principles and complete reporting, as described under “Subgroup analysis and investigation of heterogeneity “.

Meta-analyses will be undertaken where meaningful; if the participants, type of intervention and study design are homogenous enough to warrant pooling. We anticipate only continuous data to be reported for the outcomes of interest. Continuous data will be analysed as the mean difference (MD) where outcome data are reported on a uniform scale or standardized mean difference (SMD) when different metrics are used but deemed clinically homogenous. Meta-analyses will be based on change from

For peer review only - http://bmjopen.bmj.com/site/about/guidelines.xhtml

baseline rather than post-intervention values when available. Data extraction, and any needed imputations or will be done according to recommendations in Cochrane Handbook for Systematic Reviews of Interventions Version 6.4. If needed, additional information from the study authors will be sought using e-mail. A random-effects model will be used to allow for random error and inter-study variability.

We will use per-protocol analyses as the effect of interest, where they are reported. If only intention-to-treat data is reported, this will be used for analysis. If both per-protocol and intention-to-treat data are reported, the intention-to-treat data will be used in a sensitivity analysis. Where multiple trial arms are reported in a single study, we will include only relevant ones. If applicable, we will combine intervention arms or reported subgroups as per the recommendations in Cochrane Handbook for Systematic Reviews of Interventions Version 6.4.

Assessment of heterogeneity

Studies included in the review are likely to be methodologically diverse. As such, heterogeneity in the results is to be expected. We will use the I^2 and τ^2 statistics to measure heterogeneity between the studies in each analysis. If we identify substantial heterogeneity ($I^2 \geq 50\%$), we will report it and explore the possible causes by prespecified subgroup analysis. The importance of the I^2 statistic will be interpreted together with the confidence interval and p-value from the $\chi 2$ statistic, as well as the magnitude and direction of effect as described in the in Cochrane Handbook for Systematic Reviews of Interventions Version 6.4.

Assessment of reporting biases

Publication bias threatens the validity of a review's findings. To address this, if we can pool more than 10 studies, we will create and examine the asymmetry of a funnel plot and perform Egger's test to explore possible small study and publication biases. The Funnel plot provides visual aid for detection of publication bias, indicating whether there is a lack of studies reporting negative or insignificant results.

To further investigate and adjust for publication bias, we will employ the trim-and-fill method[39]. This method is designed to identify and correct for asymmetries in the funnel plot that suggest the presence of publication bias. In the event of funnel plot asymmetry, we will use the trim-and-fill method to impute ostensibly missing effect sizes as a sensitivity analysis. Grading of Recommendations Assessment, Development, and Evaluation (GRADE) is used for assessments of the quality of the evidence.

Subgroup analysis and investigation of heterogeneity

We plan to conduct subgroup analyses to investigate the effect of application of training principles and complete reporting on intervention efficacy:

- Applying relevant principles versus not applying relevant principles: This subgroup analysis will be performed per principle.
- Complete or almost complete reporting of training variables (defined as reporting ≥85% of relevant variables, e.g., eight out of nine variables) versus non-complete reporting of training variables (defined as reporting <85% relevant variables)
- High versus low compliance with CERT items. No accepted cut-offs exist for compliance to the CERT. Therefore, a post-hoc decision will be made on which cut-off to use to enable an adequate comparison with reasonable subgroups.
- Studies published after versus before publication of CERT.

For peer review only - http://bmjopen.bmj.com/site/about/guidelines.xhtml

For peer review only

Other planned subgroup analyses, not related to training principles or complete reporting but important effect modifiers, which will be performed if an adequate number of studies per analysis is available:

- Sex.
- Exercise intensity.
- Duration of intervention (≥15 sessions compared with <15 sessions).
- Severity of COPD (GOLD A and B versus C and D or E).

Additional sub-group analyses may be added if an adequate number of studies per analysis is available. Any sub-analysis that is not pre-specified will be clearly presented as such and presented in a supplementary material.

To further investigate if methodological diversity moderates the effect on the selected outcomes, a meta-regression will be performed. A meta-regression is useful when there is a wide range of values in moderator variables but few studies with the exact same value for that moderator. We expect a wide range in moderator variables such as number of CERT items reported, or training principles applied.

We will employ a random-effects univariate meta-regression to examine the relationship between the extent of complete reporting, application of training principles and change in outcomes[40].

The dependent variable in the meta-regression will be the mean difference in a continuous outcome (for example, change in 6MWD). We will examine how this effect estimate is influenced by explanatory variables, such as number of applied training principles or compliance with CERT (number of items). As such, the meta-regression model aims to predict the study's effect size, based on characteristics of the studies.

Sensitivity analysis

We plan to carry out the following sensitivity analyses, removing the following from the primary outcome analyses to examine whether findings are robust to potentially influential decisions:

- Trials judged in the quality assessment to be of low quality.
- Trials with low or no compliance to training principles, defined as applying zero, one or two (out of eight) training principles.
- Trials reporting a low number of training variables (defined as reporting 33% or less of relevant variables).
- Trials with low compliance with the CERT checklist. No generally accepted cut-offs exist for compliance to the CERT. Therefore, a post-hoc decision will be made on which cut-off to use to enable an adequate sensitivity analysis.

We will compare results from a fixed-effect model versus a random-effect model. If we impute SD using correlation coefficients in the synthesis, sensitivity analyses will be performed with different values of the correlation coefficient to determine if the overall results are robust to the imputed correlation coefficients[40]. The sensitivity analyses will be presented in a summary table. Other suitable sensitivity analyses, as identified during the review process, may also be performed, and reported.

Assessment of the certainty of the evidence

We will employ GRADE approach to assess the certainty of the body of evidence related to the included primary and secondary outcomes. The GRADE approach includes assessing evidence across several key

For peer review only

For peer review only - http://bmjopen.bmj.com/site/about/guidelines.xhtml

dimensions: Risk of bias, imprecision, inconsistency, indirectness, and publication bias. Thus, the quality of the evidence will be graded as 'high', 'moderate', 'low' or 'very low'. We will use the methods and recommendations described in the Cochrane Handbook for Systematic Reviews of Interventions V.6.4 using the GRADEpro software.

Patient and public involvement

None.

ETHICS AND DISSEMINATION

As this study is a systematic review, ethical approval is not required. The systematic review will be submitted for publication in a peer-reviewed journal. We also intend to present the findings at national and international conferences and inform patient organisations.

DISCUSSION

This systematic review is designed to critically assess the application of exercise training principles and the completeness of reporting in RCTs with an aerobic training intervention in the COPD literature. This is, to our knowledge, the first systematic review addressing this topic in COPD trials.

The findings of this systematic review are expected to indicate shortcomings in aerobic training interventions and reporting of RCTs in COPD. Based on the findings, we will provide recommendations for future work to enhance the quality of exercise prescription as well as study quality and reporting practices. Identified limitations or strengths in the included studies could inform future guidelines revisions. This, in turn, might lead to more tailored and effective exercise training in pulmonary rehabilitation.

Registration of the systematic review and publication of this protocol promotes transparency in the review process. It also minimises the risk of duplication of similar reviews. We strive for a high methodological standard by adhering to PRISMA-P, and SWiM guidelines. This review is not without limitations. The exclusion of literature written in other languages than English might leave relevant literature out of the systematic review. For this reason, we will report the number of publications that are excluded due to this reason.

Amendments

We will conduct the review according to this protocol and justify any deviations in a specific section of the systematic review.

Author Contributions

JJ contributed to the design of the study and the protocol, developed the data synthesis and meta-analysis, and wrote the initial version of the manuscript.

AS contributed to the design of the study and the protocol, developed the search strategy, data extraction and data synthesis process.

JJ and AS contributed equally as co-first authors.

HvH contributed to conceptualising the study, the design of the protocol and reviewed the protocol critically for important intellectual content.

JDB contributed to the design of the protocol, developed the data synthesis and meta-analysis, and reviewed the protocol critically for important intellectual content.

For peer review only

AN contributed to conceptualising the study, the design of the protocol, developed the data synthesis and meta-analysis, and reviewed the protocol critically for important intellectual content.

PK conceptualised the study, developed the data synthesis and meta-analysis, as the guarantor for the protocol and reviewed the protocol critically for important intellectual content.

AN and PK contributed equally as co-senior authors.

All authors read, approved, and contributed to the final written manuscript.

Funding

This work is undertaken as part of JJ's PhD work, funded by the Swedish Research Council (Vetenskapsrådet), grant number: 2020-01296.

AS was financially supported by Lung Foundation [#5.1.18.232], the Netherlands.

Funders have had no role in the design of this protocol or in the systematic review; nor in the decision to report and publish the results.

Non-financial support has been provided by the library at Radboud University, in developing the search strategy.

Competing interests

None declared.

Patient consent

Not applicable.

For peer review only

References

1. World Health Organization. The top 10 causes of death World Health Organization; 2020 [cited 2021. Available from: https://www.who.int/news-room/fact-sheets/detail/the-top-10-causes-of-death accessed 2021-09-07 2021.
2. Global Initiative for Chronic Obstructive Lung Disease. Global Strategy for the Diagnosis, Management, and Prevention of Chronic Obstructive Pulmonary Disease 2023 Report 2022 [cited 2023 2022-01-31]. accessed 2023-01-31 2023.
3. Spruit MA, Singh SJ, Garvey C, et al. An official American Thoracic Society/European Respiratory Society statement: key concepts and advances in pulmonary rehabilitation. *Am J Respir Crit Care Med* 2013;188(8):e13-64. doi: 10.1164/rccm.201309-1634ST [published Online First: 2013/10/17]
4. Gloeckl R, Zwick RH, Fürlinger U, et al. Prescribing and adjusting exercise training in chronic respiratory diseases - Expert-based practical recommendations. *Pulmonology* 2023;29(4):306-14. doi: 10.1016/j.pulmoe.2022.09.004 [published Online First: 20221020]
5. Rochester CL, Alison JA, Carlin B, et al. Pulmonary Rehabilitation for Adults with Chronic Respiratory Disease: An Official American Thoracic Society Clinical Practice Guideline. *Am J Respir Crit Care Med* 2023;208(4):e7-e26. doi: 10.1164/rccm.202306-1066ST
6. Ward TJC, Plumptre CD, Dolmage TE, et al. Change in $\dot{V}O_2$peak in Response to Aerobic Exercise Training and the Relationship With Exercise Prescription in People With COPD: A Systematic Review and Meta-analysis. *Chest* 2020;158(1):131-44. doi: https://doi.org/10.1016/j.chest.2020.01.053
7. Higashimoto Y, Ando M, Sano A, et al. Effect of pulmonary rehabilitation programs including lower limb endurance training on dyspnea in stable COPD: A systematic review and meta-analysis. *Respir Investig* 2020;58(5):355-66. doi: 10.1016/j.resinv.2020.05.010 [published Online First: 20200711]
8. Ryrsø CK, Godtfredsen NS, Kofod LM, et al. Lower mortality after early supervised pulmonary rehabilitation following COPD-exacerbations: a systematic review and meta-analysis. *BMC Pulm Med* 2018;18(1):154. doi: 10.1186/s12890-018-0718-1
9. Paneroni M, Simonelli C, Vitacca M, et al. Aerobic Exercise Training in Very Severe Chronic Obstructive Pulmonary Disease: A Systematic Review and Meta-Analysis. *Am J Phys Med Rehabil* 2017;96(8):541-48. doi: 10.1097/phm.0000000000000667
10. Gloeckl R, Marinov B, Pitta F. Practical recommendations for exercise training in patients with COPD. *Eur Respir Rev* 2013;22(128):178-86. doi: 10.1183/09059180.00000513
11. Begg C, Cho M, Eastwood S, et al. Improving the quality of reporting of randomized controlled trials. The CONSORT statement. *JAMA* 1996;276(8):637-9. doi: 10.1001/jama.276.8.637
12. Schulz KF, Altman DG, Moher D. CONSORT 2010 statement: updated guidelines for reporting parallel group randomised trials. *BMJ* 2010;340:c332. doi: 10.1136/bmj.c332 [published Online First: 2010/03/25]
13. Glasziou P, Altman DG, Bossuyt P, et al. Reducing waste from incomplete or unusable reports of biomedical research. *Lancet* 2014;383(9913):267-76. doi: 10.1016/s0140-6736(13)62228-x [published Online First: 20140108]
14. Arienti C, Armijo-Olivo S, Minozzi S, et al. Methodological Issues in Rehabilitation Research: A Scoping Review. *Arch Phys Med Rehabil* 2021;102(8):1614-22.e14. doi: 10.1016/j.apmr.2021.04.006 [published Online First: 20210511]
15. Slade SC, Dionne CE, Underwood M, et al. Consensus on Exercise Reporting Template (CERT): Explanation and Elaboration Statement. *Br J Sports Med* 2016;50(23):1428-37. doi: 10.1136/bjsports-2016-096651 [published Online First: 2016/10/07]
16. Mesquida C, Murphy J, Lakens D, et al. Replication concerns in sports and exercise science: a narrative review of selected methodological issues in the field. *Royal Society Open Science* 2022;9(12):220946. doi: 10.1098/rsos.220946

For peer review only

For peer review only - http://bmjopen.bmj.com/site/about/guidelines.xhtml

17. Hoogeboom TJ, Kousemaker MC, van Meeteren NL, et al. i-CONTENT tool for assessing therapeutic quality of exercise programs employed in randomised clinical trials. *Br J Sports Med* 2020 doi: 10.1136/bjsports-2019-101630 [published Online First: 2020/11/05]

18. Moecke DP, Zhu K, Gill J, et al. A systematic review of exercise studies for individuals hospitalized with an acute exacerbation of chronic obstructive pulmonary disease: Focus on the principles of exercise training. *Chron Respir Dis* 2023;20:14799731231215363. doi: 10.1177/14799731231215363

19. Westra B, de Wolf S, Bij de Vaate E, et al. Quality of resistance training description in COPD trials: study protocol for a systematic review. *BMJ open* 2019;9(1):e025030. doi: https://dx.doi.org/10.1136/bmjopen-2018-025030

20. Holden S, Rathleff MS, Jensen MB, et al. How can we implement exercise therapy for patellofemoral pain if we don't know what was prescribed? A systematic review. *Br J Sports Med* 2018;52(6):385. doi: 10.1136/bjsports-2017-097547 [published Online First: 20171030]

21. Jo D, Del Bel MJ, McEwen D, et al. A study of the description of exercise programs evaluated in randomized controlled trials involving people with fibromyalgia using different reporting tools, and validity of the tools related to pain relief. *Clin Rehabil* 2019;33(3):557-63. doi: 10.1177/0269215518815931 [published Online First: 2018/12/06]

22. Medysky ME, Bland KA, Neil-Sztramko SE, et al. Attention to the Principles of Exercise Training in Exercise Studies of Persons With Lung Cancer: A Systematic Review. *J Aging Phys Act* 2021:1-11. doi: 10.1123/japa.2020-0269 [published Online First: 2021/05/09]

23. Neil-Sztramko SE, Medysky ME, Campbell KL, et al. Attention to the principles of exercise training in exercise studies on prostate cancer survivors: a systematic review. *BMC Cancer* 2019;19(1):321. doi: 10.1186/s12885-019-5520-9 [published Online First: 2019/04/07]

24. Neil-Sztramko SE, Winters-Stone KM, Bland KA, et al. Updated systematic review of exercise studies in breast cancer survivors: attention to the principles of exercise training. *Br J Sports Med* 2019;53(8):504-12. doi: 10.1136/bjsports-2017-098389 [published Online First: 2017/11/23]

25. Hoffman J. Physiological Aspects of Sport Training and Performance. Champaign: IL: Human Kinetics 2002.

26. Kasper K. Sports Training Principles. *Curr Sports Med Rep* 2019;18(4)

27. Campbell KL, Neil SE, Winters-Stone KM. Review of exercise studies in breast cancer survivors: attention to principles of exercise training. *Br J Sports Med* 2012;46(13):909-16. doi: 10.1136/bjsm.2010.082719

10.1136/bjsports-2010-082719 [published Online First: 2012/09/26]

28. Thomas JH, Martijn CK, Nico LUvM, et al. i-CONTENT tool for assessing therapeutic quality of exercise programs employed in randomised clinical trials. *Br J Sports Med* 2021;55(20):1153. doi: 10.1136/bjsports-2019-101630

29. McCarthy B, Casey D, Devane D, et al. Pulmonary rehabilitation for chronic obstructive pulmonary disease. *Cochrane Database Syst Rev* 2015(2):Cd003793. doi: 10.1002/14651858.CD003793.pub3 [published Online First: 2015/02/24]

30. Page MJ, McKenzie JE, Bossuyt PM, et al. The PRISMA 2020 statement: an updated guideline for reporting systematic reviews. *BMJ* 2021;372:n71. doi: 10.1136/bmj.n71

31. Nici L, Donner C, Wouters E, et al. American Thoracic Society/European Respiratory Society statement on pulmonary rehabilitation. *Am J Respir Crit Care Med* 2006;173(12):1390-413. doi: 10.1164/rccm.200508-1211ST [published Online First: 2006/06/09]

32. Ouzzani M, Hammady H, Fedorowicz Z, et al. Rayyan—a web and mobile app for systematic reviews. *Systematic Reviews* 2016;5(1):210. doi: 10.1186/s13643-016-0384-4

33. Bland KA, Neil-Sztramko SE, Zadravec K, et al. Attention to principles of exercise training: an updated systematic review of randomized controlled trials in cancers other than breast and prostate. *BMC Cancer* 2021;21(1):1179. doi: 10.1186/s12885-021-08701-y

For peer review only - http://bmjopen.bmj.com/site/about/guidelines.xhtml

669 34. Winters-Stone KM, Neil SE, Campbell KL. Attention to principles of exercise training: a review of exercise studies for survivors of cancers other than breast. *Br J Sports Med* 2014;48(12):987-95. doi: 10.1136/bjsports-2012-091732 [published Online First: 2013/01/08]
672 35. Nyberg A, Probst V, Vaes AW. Assessments. In: Holland AE, Corso SD, Spruit MA, eds. Pulmonary Rehabilitation: European Respiratory Society 2021:360.
674 36. Smart NA, Waldron M, Ismail H, et al. Validation of a new tool for the assessment of study quality and reporting in exercise training studies: TESTEX. *Int J Evid Based Healthc* 2015;13(1):9-18. doi: 10.1097/xeb.0000000000000020
677 37. Stefanakis M, Batalik L, Antoniou V, et al. Safety of home-based cardiac rehabilitation: A systematic review. *Heart Lung* 2022;55:117-26. doi: https://doi.org/10.1016/j.hrtlng.2022.04.016
679 38. Campbell M, McKenzie JE, Sowden A, et al. Synthesis without meta-analysis (SWiM) in systematic reviews: reporting guideline. *BMJ* 2020;368:l6890. doi: 10.1136/bmj.l6890
681 39. Duval S, Tweedie R. Trim and Fill: A Simple Funnel-Plot-Based Method of Testing and Adjusting for Publication Bias in Meta-Analysis. *Biometrics* 2000;56(2):455-63.
683 40. Higgins JPT TJ, Chandler J, Cumpston M, Li T, Page MJ, Welch VA (editors). Cochrane Handbook for Systematic Reviews of Interventions version 6.2 (updated February 2021): Cochrane, 2021, 2021.

For peer review only

Reporting checklist for protocol of a systematic review and meta analysis.

Based on the PRISMA-P guidelines.

Instructions to authors

Complete this checklist by entering the page numbers from your manuscript where readers will find each of the items listed below.

Your article may not currently address all the items on the checklist. Please modify your text to include the missing information. If you are certain that an item does not apply, please write "n/a" and provide a short explanation.

Upload your completed checklist as an extra file when you submit to a journal.

In your methods section, say that you used the PRISMA-Preporting guidelines, and cite them as:

Moher D, Shamseer L, Clarke M, Ghersi D, Liberati A, Petticrew M, Shekelle P, Stewart LA. Preferred Reporting Items for Systematic Review and Meta-Analysis Protocols (PRISMA-P) 2015 statement. Syst Rev. 2015;4(1):1.

		Reporting Item	Page Number
**Title**			
Identification	#1a	Identify the report as a protocol of a systematic review	1
Update	#1b	If the protocol is for an update of a previous systematic review, identify as such	NA
**Registration**			
	#2	If registered, provide the name of the registry (such as PROSPERO) and registration number	2
**Authors**			
Contact	#3a	Provide name, institutional affiliation, e-mail address of all protocol authors; provide physical mailing address of corresponding author	1
Contribution	#3b	Describe contributions of protocol authors and identify the	17

For peer review

		guarantor of the review	
**Amendments**			
	#4	If the protocol represents an amendment of a previously completed or published protocol, identify as such and list changes; otherwise, state plan for documenting important protocol amendments	16
**Support**			
Sources	#5a	Indicate sources of financial or other support for the review	17
Sponsor	#5b	Provide name for the review funder and / or sponsor	NA
Role of sponsor or funder	#5c	Describe roles of funder(s), sponsor(s), and / or institution(s), if any, in developing the protocol	17
**Introduction**			
Rationale	#6	Describe the rationale for the review in the context of what is already known	4-5
Objectives	#7	Provide an explicit statement of the question(s) the review will address with reference to participants, interventions, comparators, and outcomes (PICO)	5
**Methods**			
Eligibility criteria	#8	Specify the study characteristics (such as PICO, study design, setting, time frame) and report characteristics (such as years considered, language, publication status) to be used as criteria for eligibility for the review	5-6
Information sources	#9	Describe all intended information sources (such as electronic databases, contact with study authors, trial registers or other grey literature sources) with planned dates of coverage	6-7
Search strategy	#10	Present draft of search strategy to be used for at least one electronic database, including planned limits, such that it could be repeated	7 and appendix
Study records - data management	#11a	Describe the mechanism(s) that will be used to manage records and data throughout the review	7-8

For peer review only

Study records - selection process	#11b	State the process that will be used for selecting studies (such as two independent reviewers) through each phase of the review (that is, screening, eligibility and inclusion in meta-analysis)	7
Study records - data collection process	#11c	Describe planned method of extracting data from reports (such as piloting forms, done independently, in duplicate), any processes for obtaining and confirming data from investigators	8-12
Data items	#12	List and define all variables for which data will be sought (such as PICO items, funding sources), any pre-planned data assumptions and simplifications	8-12
Outcomes and prioritization	#13	List and define all outcomes for which data will be sought, including prioritization of main and additional outcomes, with rationale	12
Risk of bias in individual studies	#14	Describe anticipated methods for assessing risk of bias of individual studies, including whether this will be done at the outcome or study level, or both; state how this information will be used in data synthesis	12-13 + 15
Data synthesis	#15a	Describe criteria under which study data will be quantitatively synthesised	14-15
Data synthesis	#15b	If data are appropriate for quantitative synthesis, describe planned summary measures, methods of handling data and methods of combining data from studies, including any planned exploration of consistency (such as I2, Kendall's τ)	14-15
Data synthesis	#15c	Describe any proposed additional analyses (such as sensitivity or subgroup analyses, meta-regression)	14-15
Data synthesis	#15d	If quantitative synthesis is not appropriate, describe the type of summary planned	13-14
Meta-bias(es)	#16	Specify any planned assessment of meta-bias(es) (such as publication bias across studies, selective reporting within studies)	14
Confidence in cumulative evidence	#17	Describe how the strength of the body of evidence will be assessed (such as GRADE)	15-16

For peer review only

Notes:

- 10: 8 and appendix The PRISMA-P elaboration and explanation paper is distributed under the terms of the Creative Commons Attribution License CC-BY. This checklist was completed on 12. January 2024 using https://www.goodreports.org/, a tool made by the EQUATOR Network in collaboration with Penelope.ai

For peer review only

Online appendix 2. Search strategies

The number within brackets after every search terms is the number of hits for that search.

1. Search strategy for PubMed

Date: 27-05-2021

#1:

"Pulmonary Disease, Chronic Obstructive"[Mesh] OR Chronic Obstructive Pulmonary Disease* [tiab] OR COPD [tiab] OR COAD [tiab] OR Chronic Obstructive Airway Disease* [tiab] OR Chronic Obstructive Lung Disease* [tiab] OR chronic airflow obstruction* [tiab] OR chronic airflow limitation*[tiab] OR Emphysema[tiab] OR Chronic bronchitis[tiab] OR chronic obstructive bronchitis[tiab] OR chronic obstructive bronchopulmonary disease*[tiab] OR chronic obstructive lung disorder*[tiab] OR chronic obstructive pulmonary disorder*[tiab] OR chronic obstructive respiratory disease*[tiab] OR lung chronic obstructive disease*[tiab] (111 965)

#2:

"Pulmonary Disease, Chronic Obstructive/rehabilitation"[Mesh] OR "Rehabilitation"[Mesh:NoExp] OR Exercise Therapy[Mesh] OR "Exercise"[Mesh] OR "Bicycling"[Mesh] OR Aerobic training* [tiab] OR aerobic exercise*[tiab] OR exercise training*[tiab] OR rehabilitation[tiab] OR Exercise Therap*[tiab] OR Endurance Training*[tiab] OR Walking[tiab] OR swimming[tiab] OR rowing[tiab] OR endurance exercise*[tiab] OR interval training*[tiab] OR cycling[tiab] OR bicycling[tiab] OR training*[tiab] OR hit[tiab] (941 554)

#3: #1 AND #2 (9 021)

#4: #1 AND #2 Filters: English (7 931)

#5: #1 AND #2 Filters: from 2007 – 3000/12/12 (6 344)

#6: #1 AND #2 Filters: English, from 2007 – 3000/12/12 (5 828)

Total hits: 5 828

For peer review only

Begränsad delning

2. Search strategy for Embase

Date: 27-05-2021

1. *chronic obstructive lung disease/ or *lung emphysema/ or *chronic bronchitis/ or (Chronic Obstructive Pulmonary Disease* or COPD or COAD or Chronic Obstructive Airway Disease* or Chronic Obstructive Lung Disease* or chronic airflow obstruction* or chronic airflow limitation* or Emphysema or Chronic bronchitis or chronic obstructive bronchitis or chronic obstructive bronchopulmonary disease* or chronic obstructive lung disorder* or chronic obstructive pulmonary disorder* or chronic obstructive respiratory disease* or lung chronic obstructive disease*).ti,ab,kw. (170 046)

2. chronic obstructive lung disease/rh or lung emphysema/rh or chronic bronchitis/rh or exercise/ or aerobic exercise/ or endurance training/ or high intensity interval training/ or training/ or rehabilitation/ or pulmonary rehabilitation/ or kinesiotherapy/ or walking/ or swimming/ or rowing/ or cycling/ or (Aerobic training* or aerobic exercise* or exercise training* or rehabilitat* or Exercise Therap* or Endurance Training* or Walking or swimming or rowing or endurance exercise* or interval training* or cycling or bicycling or training* or hit).ti,ab,kw. (1 349 026)

3. 1 and 2 (18 989)

4. limit 3 to english language (16 992)

5. limit 3 to yr="2007 -Current" (14 531)

6. limit 3 to (english language and yr="2007 -Current") (13 740)

7. limit 6 to conference abstract status (6 130)

8. 6 not 7 (7 610)

Total hits: 7 610

For peer review only

3. Search strategy for Cinahl

Date: 27-05-2021

S1. (MH "Pulmonary Disease, Chronic Obstructive+") OR (MH "Emphysema") OR TI ("Chronic Obstructive Pulmonary Disease*" OR COPD OR COAD OR "Chronic Obstructive Airway Disease*" OR "Chronic Obstructive Lung Disease*" OR "chronic airflow obstruction*" OR "chronic airflow limitation*" OR Emphysema OR "Chronic bronchitis" OR "chronic obstructive bronchitis" OR "chronic obstructive bronchopulmonary disease*" OR "chronic obstructive lung disorder*" OR "chronic obstructive pulmonary disorder*" OR "chronic obstructive respiratory disease*" OR "lung chronic obstructive disease*") OR AB ("Chronic Obstructive Pulmonary Disease*" OR COPD OR COAD OR "Chronic Obstructive Airway Disease*" OR "Chronic Obstructive Lung Disease*" OR "chronic airflow obstruction*" OR "chronic airflow limitation*" OR Emphysema OR "Chronic bronchitis" OR "chronic obstructive bronchitis" OR "chronic obstructive bronchopulmonary disease*" OR "chronic obstructive lung disorder*" OR "chronic obstructive pulmonary disorder*" OR "chronic obstructive respiratory disease*" OR "lung chronic obstructive disease*") (33 553)

Expanders: Apply equivalent subjects

Search modes: Boolean/Phrase

S2. (MH "Pulmonary Disease, Chronic Obstructive+/RH") OR (MH "Emphysema/RH") OR (MH "Exercise+") OR (MH "Physical Therapy+") OR (MH "Rehabilitation") OR (MH "Rehabilitation, Pulmonary+") OR (MH "Swimming") OR (MH "Rowing") OR (MH "Cycling") OR TI ("Aerobic training*" OR "aerobic exercise*" OR "exercise training*" OR rehabilitat* OR "Exercise Therap*" OR "Endurance Training*" OR Walking OR swimming OR rowing OR "endurance exercise*" OR "interval training*" OR cycling OR bicycling OR training* OR hit) OR AB ("Aerobic training*" OR "aerobic exercise*" OR "exercise training*" OR rehabilitat* OR "Exercise Therap*" OR "Endurance Training*" OR Walking OR swimming OR rowing OR "endurance exercise*" OR "interval training*" OR cycling OR bicycling OR training* OR hit) (511 922)

Expanders: Apply equivalent subjects

Search modes: Boolean/Phrase

S3. S1 AND S2 (4 398)

Expanders: Apply equivalent subjects

Search modes: Boolean/Phrase

S4. S1 AND S2. (4 147)

Expanders: Apply equivalent subjects

Search modes: Boolean/Phrase

Narrow by Language: English

S5. S1 AND S2 (3 344)

Limiters: Published Date: 20070101-20211231

For peer review only

Search modes: Boolean/Phrase

Narrow by Language: English

S6. S1 and S2 (3 130)

Limiters: Published Date: 20070101-20211231

Search modes: Boolean/Phrase

Narrow by Language: English

Expanders – Apply equivalent subjects

Total hits: 3 130

4. Cochrane search strategy

Date: 27-05-2021

Expanders: Word variations enabled.

#1: ("Chronic Obstructive Pulmonary Disease" OR COPD OR COAD OR "Chronic Obstructive Airway Disease" OR "Chronic Obstructive Lung Disease" OR "chronic airflow obstruction" OR "chronic airflow limitation" OR Emphysema OR "Chronic bronchitis" OR "chronic obstructive bronchitis" OR "chronic obstructive bronchopulmonary disease" OR "chronic obstructive lung disorder" OR "chronic obstructive pulmonary disorder" OR "chronic obstructive respiratory disease" OR "lung chronic obstructive disease") :ti,ab,kw

#2: ("Aerobic training" OR "aerobic exercise" OR "exercise training" OR rehabilitat* OR "Exercise Therapy" OR "Endurance Training" OR Walking OR swimming OR rowing OR "endurance exercise" OR "interval training" OR cycling OR bicycling OR training OR hit) :ti,ab,kw

#3: #1 AND #2

Cochrane reviews: 38

Trials: 5415

Cochrane protocols: 8, not included

Editorials: 2, not included

Clinical Answers: 2, not included

Total hits: 5453

For peer review only

5. PEDro search strategy

Date: 28-05-2021

#1. Chronic respiratory disease (topic) + aerobic training (abstract and title) (86)

- Limit to systematic reviews and clinical trials (84)
- Other chronic respiratory diseases than COPD excluded (33):
- Remaining hits: 51
- Other languages removed (4)

Total records remaining: 47

For peer review only

BMJ Open

Quality of aerobic training description and its relation to intervention efficacy in chronic obstructive pulmonary disease trials: study protocol for a systematic review, meta-analysis, and meta-regression

Journal:	*BMJ Open*
Manuscript ID	bmjopen-2024-084296.R2
Article Type:	Protocol
Date Submitted by the Author:	10-May-2024
Complete List of Authors:	Jakobsson, Johan; Umea University, Community Medicine and Rehabilitation, Physiotherapy<br>Stoffels, Anouk ; Radboud Universiteit, Department of Pulmonary Diseases<br>van Hees, Hieronymus; Radboud Universiteit, Department of Pulmonary Diseases<br>De Brandt, Jana; Umeå Universitet, Department of Community Medicine and Rehabilitation, Physiotherapy<br>Nyberg, André; Umeå University, Department of Community Medicine and Rehabilitation, Physiotherapy<br>Klijn, Peter; Merem Pulmonary Rehabilitation Center; Amsterdam University Medical Centres, Department of Pulmonary Medicine
**Primary Subject Heading**:	Respiratory medicine
Secondary Subject Heading:	Sports and exercise medicine, Rehabilitation medicine, Public health
Keywords:	Pulmonary Disease < Lung Diseases, Pulmonary Disease, Chronic Obstructive, SPORTS MEDICINE, REHABILITATION MEDICINE, Physical Therapy Modalities

SCHOLARONE™
Manuscripts

Quality of aerobic training description and its relation to intervention efficacy in chronic obstructive pulmonary disease trials: study protocol for a systematic review, meta-analysis, and meta-regression

Authors

Johan Jakobsson[1]*#, Anouk A.F. Stoffels#[2], Hieronymus W.H. van Hees[2], Jana De Brandt[1], Andre Nyberg§[1], Peter Klijn§[3, 4]

Affiliations

[1]Umeå University, Department of Community Medicine and Rehabilitation, Section of Physiotherapy, Umeå, Sweden.

[2]Department of Pulmonary Diseases, Radboud University Medical Center, Nijmegen, the Netherlands.

[3]Merem Pulmonary Rehabilitation Center, Hilversum, the Netherlands.

[4]Department of Pulmonary Medicine, Amsterdam University Medical Centre, Amsterdam, the Netherlands

#Authors contributed equally as joint first authors.

§Authors contributed equally as senior authors.

*Correspondence to:

Johan Jakobsson, Department of Community Medicine and Rehabilitation, Section of Physiotherapy, SE-901 87, Universitetstorget 4, Sweden
johan.jakobsson@umu.se

Emails:

Hieronymus W.H. van Hees: Jeroen.vanHees@radboudumc.nl

Anouk A. F. Stoffels: a_stoffels@hotmail.com

Jana De Brandt: jana.de.brandt@umu.se

Andre Nyberg: andre.nyberg@umu.se

Peter Klijn: PKlijn@merem.nl

For peer review only

For peer review only - http://bmjopen.bmj.com/site/about/guidelines.xhtml

Word count: 4964 (Excluding abstract, tables, references)

Keywords: COPD; Exercise training; pulmonary rehabilitation; pulmonary disease, chronic obstructive; Exercise prescription

For peer review only

For peer review only - http://bmjopen.bmj.com/site/about/guidelines.xhtml

Abstract

Introduction: Chronic obstructive pulmonary disease (COPD) is a major global health concern, characterized by ventilatory constraints, decreased cardiovascular fitness and reduced limb muscle function, profoundly affecting patients' quality of life. Aerobic training plays a crucial role in the treatment of COPD, but the variability in methodologies and incomplete reporting of key components in aerobic training trials limits the assessment of their effectiveness. This systematic review aims to critically evaluate the application of training principles and reporting of key components in aerobic training trials in randomized controlled trials (RCTs) in the COPD literature.

Methods and analysis: The protocol adheres to the Preferred Reporting Items for Systematic Reviews and Meta Analyses Protocol guidelines. The review will include RCTs utilizing aerobic training in individuals with COPD. A comprehensive search, following a predefined search strategy will identify studies published from 2007 to 2024 in English from MEDLINE, Embase, CINAHL, CENTRAL and PEDro. Studies including people with COPD and any aerobic training intervention will be included. Two reviewers will independently screen abstracts and titles for inclusion. Two reviewers will independently conduct the screening of full-text documents and data extraction. Study quality will be assessed using the Tool for the assESsment of sTudy quality and bias in Exercise, specifically developed for exercise training studies. The certainty of the evidence will be evaluated using the Grading of Recommendations Assessment, Development and Evaluation approach. A systematic synthesis will be provided, with meta-analyses and meta-regression when appropriate.

Ethics and dissemination: As this review will involve the analysis of published data, ethical approval is not required. The findings of this review will be disseminated through peer-reviewed publications and conference presentations.

Study registration: PROSPERO, CRD42021247343.

For peer review only

For peer review only - http://bmjopen.bmj.com/site/about/guidelines.xhtml

Strengths and limitations of this study

- The protocol includes a comprehensive search strategy and broad inclusion criteria to allow for a comprehensive synthesis of available evidence.
- This protocol is reported according to the Preferred Reporting Items for Systematic Reviews and Meta-Analyses Protocols.
- We will examine the quality of included studies using the Tool for the assESsment of sTudy quality and bias in EXercise specifically developed for trials with exercise interventions.
- Exclusion of literature written in languages not known by the research group might leave relevant literature out of the systematic review.

For peer review only

For peer review only - http://bmjopen.bmj.com/site/about/guidelines.xhtml

INTRODUCTION

Chronic obstructive pulmonary disease (COPD) stands as the third leading cause of death[1], with more than 384 million individuals diagnosed globally[2]. In the management of COPD, the beneficial impacts of pulmonary rehabilitation, incorporating aerobic exercise training as a key element, are well-documented[2-5]. These improvements typically manifest in increased cardiorespiratory fitness[6], better overall quality of life and it reduces the burden of symptoms[3 7-9]. In fact, among different exercises modalities within PR, aerobic training is the most frequently applied and investigated mode of exercise in COPD[3 10], often investigated using a randomised controlled trial (RCT).

However, the reporting of RCTs is often incomplete, giving rise to the Consolidated Standards of Reporting Trials (CONSORT) statement in 1996[11]. Provided that the field of medicine depends on transparent reporting of RCTs[12], inadequate reporting of trials is troublesome. As a result, the reliability and validity of findings cannot be judged, nor data synthesised for systematic reviews and meta-analyses[13]. Of course, the literature of rehabilitation research and exercise training is not an exception from this[14]. Indeed, exercise training interventions are often poorly described[15], and the field suffers from other concerns regarding methodological rigor and problems with replication[16]. This negatively influences the quality of evidence, as recently concluded by a review investigating methodological rigour and reporting practices in rehabilitation research[14]. In addition, it also contributes to heterogeneity between pulmonary rehabilitation programs and hinders international benchmarking.

Inadequate reporting practices of exercise interventions led to the development of the Consensus on Exercise Reporting Template (CERT) in 2016[15]. As previously stated, it is almost unethical that researchers are still able, almost without any regulation, to design and test exercise interventions that likely have a low potential for being effective[17]. Adequate reporting of research, including exercise interventions, is the first step to help the reader to understand what the researcher did, assess intervention quality and effectiveness, translate research findings into clinical practice, and synthesise data in systematic reviews and meta-analyses for evidence-based guidelines. Although the efficacy of aerobic training interventions in COPD is evident, we, and others[18 19], have acknowledged a high level of heterogeneity in exercise training protocols and methodological rigor, as well as lacking descriptions of exercise interventions in the COPD literature. This ambiguity complicates the assessment of intervention effectiveness and challenges the replicability of these interventions and their applicability into clinical settings. As such, complete reporting of training interventions is essential. Inadequate reporting of aerobic training interventions has been reviewed previously in other chronic diseases[20-24], while trials in the COPD literature remains to be investigated.

Specifically, for exercise training trials, it is important to report all factors influencing the response to training. First, the well-established training principles[25-27] (Table 1) should be applied in designing an exercise program to ensure the most appropriate exercise type and dose to achieve the desired outcome. Secondly, important exercise training variables[15] that contribute to the training stimulus should be reported. These components should cover the FITT-principles (frequency, intensity, time, type) and information on rest, sequence of exercises and program duration[15]. Evidence on the importance of incorporating the FITT-principles within aerobic training studies are starting to emerge. Recent data suggests that FITT-principles such as frequency and time seem to be associated with an increase in peak oxygen uptake following aerobic training in individuals with COPD[6]. Thirdly, items concerning the external validity, such as eligibility criteria, setting, thorough reporting of participant characteristics, supervision and adherence to interventions must also be described[12 15 28 29]. Still, whether the efficacy of aerobic training interventions among people with COPD is related to the

For peer review only - http://bmjopen.bmj.com/site/about/guidelines.xhtml

application of exercise training principles and/or a more complete description of key exercise training variables remains to be fully determined.

Aims and objectives

We aim to investigate the application of exercise training principles and completeness of reporting in aerobic training interventions in the COPD literature. We also aim to investigate if efficacy of the interventions provided is related to the completeness of reporting and application of training principles. Our specific research questions are:

"To what extent does RCTs with an aerobic training intervention in the COPD literature apply and report the principles of exercise training, and how well are the key training variables, intervention and participant characteristics reported in these trials?

"Is the efficacy of aerobic training interventions related to the application of exercise training principles and/or a more complete description of key exercise training variables?"

METHODS AND ANALYSIS

The study protocol of this systematic review is reported in accordance with the Preferred Reporting Items for Systematic Reviews and Meta-Analyses Protocols (PRISMA-P) guideline (provided in online supplemental appendix 1). The systematic review was prospectively registered in the International Prospective Register of Systematic Reviews (PROSPERO) on 18th of May 2021 (CRD42021247343). The final review will be reported according to the PRISMA 2020 guidelines[30].

Eligibility criteria

We will select studies according to the criteria below.

Study designs

Only RCTs will be included in this systematic review. Other longitudinal study design, such as controlled non-randomized studies of interventions, cluster trials and prospective comparative cohort studies will be excluded. Studies with a cross-sectional design, retrospective studies, case series, case reports, commentaries and systematic or narrative reviews, will be excluded.

Participants

Any eligible study design with people with COPD being ≥ 90% of the participants will be considered. Studies with a higher proportion of non-COPD people can be included if the data for the COPD group is, or can be, separated. The COPD diagnosis should be based on a forced expiratory volume in one second (FEV_1) to forced vital capacity (FVC) < 0.7. There will be no eligibility criteria for disease severity, sex, age, body composition or ethnicity. Participants with additional comorbidities will not be excluded, but COPD should be the primary disease of the participants.

Interventions

Any type of aerobic training intervention of the lower limbs which is aerobically demanding is of interest. For example, cycling, walking, swimming, water-based movements, and aerobics. There will be no eligibility criteria for duration of the intervention or number of exercise sessions.

Comparators

Since this study does not intend to compare the effects between two different interventions, there will be no eligibility criteria for comparators. If there is one group with an eligible aerobic training intervention it will be included independent of type of, or the existence of, a control group in this study.

For peer review only - http://bmjopen.bmj.com/site/about/guidelines.xhtml

Outcomes

During the screening phase, studies will not be excluded based on outcomes. One objective of this systematic review is to investigate whether the key training principles are applied, and whether training components are described completely. See "data collection" for which data that will be extracted for this objective.

For the second objective, the following outcomes are eligible:

Primary outcomes

- 6-minute walking distance (6MWD, meters)
- Incremental shuttle walk test (ISWT, meters)
- Endurance shuttle walk test (ESWT, duration)
- Peak work rate (watts) or VO_2max (ml/min/kg) during cardiopulmonary exercise test (CPET)
- Constant work-rate test (duration)

Secondary outcomes

- Short physical performance battery (SPPB, score)
- Sit-to-stand tests: 5-time sit-to-stand, 30-seconds sit-to-stand or 1-minute sit-to-stand (time or reps)
- Timed-up and go test (TUG; sec)
- Stair climb power test (Watt)
- Steep ramp test (Watt)

Timing

Data from before ("baseline" or "pre"), immediately after the intervention ("post") and if applicable, additional follow-ups, will be included for intervention studies with more than two time points of outcome assessments.

Setting

There will be no eligibility criteria for type of setting. Examples of different settings could be home-based, hospital-based, inpatient, outpatient, community based or a combination of these.

Timespan

Articles from 1st of January 2007 until 2024 will be included. An initial search was done in May 2021, which will be updated prior to completion of the review. This timespan was chosen since it would include RCTs published after the first joint statement on pulmonary rehabilitation[31] by the American Thoracic Society and the European Respiratory Society in 2006.

Language

Only articles written in English will be included. A list of possibly relevant titles in other languages will be provided.

Search methods for identification of studies

Electronic searches

Studies will be identified from:

- MEDLINE (PubMed interface)
- Embase

For peer review only

For peer review only - http://bmjopen.bmj.com/site/about/guidelines.xhtml

- CINAHL
- Cochrane Central Register of Controlled Trials (CENTRAL)
- PEDro

Other resources

The reference list of includes studies will be hand-searched for other relevant studies. Also, other relevant systematic and narrative review studies will be checked for additional studies eligible for inclusion. Conference proceedings will not be included. No grey literature will be searched since we believe it will not add any additional body of literature.

The Cochrane Database of Systematic Reviews and PROSPERO have been searched for existing or ongoing reviews on the topic, with no findings.

Search strategy

To identify relevant studies, a comprehensive search will be performed. The search strategy has been developed by the authors who are researchers in respiratory physiotherapy and clinical exercise physiology, together with a librarian of the Radboud University (Nijmegen, The Netherlands). The original search strategy was developed for PubMed and has been adapted to other databases' syntax and subject headings. Comprehensive searches were constructed of both index terming (Medical subject headings (MeSH) term), free text terms and synonyms. An English language restriction was imposed on the search. The search strategy for the electronic searches, initially done in May 2021, but which will be updated in 2024, is presented in the online supplemental appendix 2.

Data collection and analysis

Study records

The results from the literature searches will be uploaded to Rayyan[32], a web-tool for collaborative systematic reviews. Screening question for selection will be pilot tested on a few papers.

The search process will be documented, including:

- the name of the database searched.
- the name of the database provider/system used.
- the date when the search was run.
- the years covered by the search.
- the search terms used, hits per search term and number of articles retrieved.

Selection process

Titles and abstracts retrieved from the searches and additional sources will be reviewed independently by two reviewers to identify potentially relevant studies for inclusion based on the eligibility criteria. Studies will be coded as “yes”, “no” or “maybe”. Studies with “no” from both reviewers will be directly excluded. Studies with two “yes” will be directly included to the next stage. Studies with conflicting decisions will be checked by at least one reviewer for inclusion or exclusion. Then, we will retrieve the full texts of all potentially eligible studies. If needed, authors will be contacted to resolve questions regarding eligibility.

Full text articles of included studies will be independently screened by pairs of reviewers for eligibility. A third reviewer will assess agreement and resolve disagreements on eligibility using a majority rule. Neither of the review authors will be blind to the journal titles, the study authors, or institutions.

For peer review only

For peer review only - http://bmjopen.bmj.com/site/about/guidelines.xhtml

The results of the selection process will be documented reported in sufficient detail to complete a PRISMA flow diagram. Missing papers will be requested from study authors using e-mail. Inter-assessor agreement will be assessed using Kappa statistics.

Data collection process

From each included study two reviewers will independently extract information on study characteristics, participants characteristics, intervention descriptions and outcome data. Data will be entered into a pilot-tested data extraction form developed a priori. All the extracted variables will then be used in the synthesis and quality appraisal of the aerobic training description. A third reviewer will assess agreement between data extractors and resolve disagreements using a majority rule. If needed, a fourth reviewer will be consulted.

The data as outlined below will be extracted. First, it will be reported whether the data is reported ("1") or not reported ("0"), if its insufficiently reported, not clear or not applicable. Secondly, the actual data will be extracted, such as the corresponding data value or a text description. Continuous data will be reported as mean ± SD or median (Q1-Q3). If it is reported otherwise, such as mean ± SEM, this will be noted. The following variables will be extracted:

- Study details: first author name, publication year, title, journal, study design, study objective, inclusion criteria, exclusion criteria and country of execution. If country of execution is not mentioned in the text, it will be derived from where ethical permission have been obtained.

Participants characteristics will be extracted and reported per intervention group. The aerobic training group will always be coded as the intervention group, and any other group will be coded as control. If the study consists of, and is reported in several subgroups, such as per disease severity, data will be reported per subgroup.

Training principles, reporting of participant characteristics, intervention characteristics and training variables will be extracted for our first objective. In addition, outcomes will be extracted for our second objective.

Participant characteristics that will be extracted:

- General
    - Sample size (n)
    - Age (years)
    - Sex distribution (number, ratio)
- Pulmonary function (coded as reported if any of the data below is provided):
    - FEV$_1$ (L)
    - FEV$_1$ (% predicted)
    - GOLD stage I, II, III or IV (number or % per group)
    - GOLD stage A, B, C or D (or A, B or E when applicable) (number or % per group)
    - FEV$_1$/FVC (ratio)
    - RV/TLC (ratio)
    - Dynamic hyperinflation (change in inspiratory capacity, % or L)
    - DLco (ml/min/kPa)
    - DLco (% predicted)
- Body composition (coded as reported if any of the data below is provided):
    - BMI (kg/m^2)
    - FFMI (kg/m^2)
    - FMI (kg/m^2)

For peer review only

For peer review only - http://bmjopen.bmj.com/site/about/guidelines.xhtml

    - Muscle mass (kg)
- Muscle strength (coded as reported if any of the data below is provided):
    - Muscle strength (kg, Nm or as appropriate)
    - Muscle strength (% predicted, with reference to predicted values)
    - Text description of apparatus and type of contraction used in the measurement.
- Exercise capacity: (coded as reported if any of the data below is provided):
    - CPET (VO_2peak mL/min/kg and % predicted, peak watt (W) and % predicted)
    - ISWT (meters, % predicted)
    - 6-MWT (meters, % predicted)
- Exercise tolerance: (coded as reported if any of the data below is provided):
    - CWRT (duration)
    - ESWT (duration)
- Training status: text description regarding previous experience or training status of participants
- Physical activity (CERT item 15): text description (i.e., sedentary, active) or as steps per day, the Physical Activity Level (PAL) or metabolic equivalents (METs).
- Smoking status: average packyears and number of participants per group of never smokers, smokers, former smokers, and non-smokers.
- Comorbidities: frequency per comorbidity, Charlson-comorbidity index or hospital anxiety and depression scale.
- Specific phenotype: text description of specific COPD-phenotypes, for example, frequent exacerbator, alpha-1-antitrypsine deficiency, asthma-COPD-overlap, amongst others.

Aerobic training intervention characteristics that will be extracted:

- Aerobic training objective: rationale regarding the benefits of the exercise program.
- Training device used, including manufacturer and type (CERT item 1). For example, only "cycle ergometer" is insufficient reporting, as type and manufacturer is missing.
- Type of exercise (CERT item 13): For example, walking, cycling, rowing, stair walking.
- Adjuncts (CERT item 10): for example, other exercise modalities, breathing exercises, nutrition interventions, medications, or education.
- Description of control intervention (CERT item 9)
- Place of aerobic training in intervention: standalone, part of pulmonary rehabilitation, as an adjunct or other.
- Supervision (CERT item 2, 4 16a): type of supervision and ratio between supervisors and participants. Only information whether it was supervised or not is insufficient.
- Supervision qualifications: experience, skills, and education of supervisors.
- Setting (CERT item 12): inpatient, outpatient, home-based, community based, hospital based, combination, or other.
- Delivery (CERT item 3): group or individual. If the delivery is in group, the group size should be specified.
- Attendance: number of, or % of, attended sessions.
- Adherence to protocol (CERT item 5, 13, 14, 16b): in addition to the number or percentage of attended sessions, information should be provided on the adherence to the prespecified protocol. Thus, whether performed intensity, duration and total load of the training was performed as planned.
- Safety: information regarding the number and type of adverse events.
- Dropouts: information regarding the number reason for drop-out.

For peer review only - http://bmjopen.bmj.com/site/about/guidelines.xhtml

Training principles that will be extracted are described in Table 1.

Table 1. Exercise training principles

Principle	Criteria used in this review	Examples and elaboration
**Progression:** Over time, adaptations in the physiological component being trained occurs. Progressions refers to the gradual and systematic increase in training stress to maintain tissue overload and thus, stimulate continued training adaptation.	Progression rules should be clearly outlined in the exercise programme, i.e. a detailed prescription of its realization. If terminology such as "as tolerated" is used, it should be clearly stated what is meant and how it is operationalized.	"The duration of the exercise session is increased by five minutes per week."<br><br>"The exercise intensity is increased by 0-10% every two weeks based on the following progression rules…"
**Overload:** For training adaptations to occur, the exposure of tissues being trained must be exercise above the accustomed-to training stress.	Rationale should be provided if exercise was prescribed adequately in relation to baseline testing or individual exercise capacity. For interventions longer than six weeks, reassessment should be performed during the intervention.	"Exercise intensity was set at 80% of maximal capacity, which was re-tested every fourth week."<br><br>When overload (intensity) is solely prescribed on baseline testing in interventions longer than six weeks, this principle is not met.
**Specificity:** The closer the training stimulus resembled the characteristic of an activity (mode of exercise, energetic system, muscles trained) the better the training adaptations will be.	The purpose of the exercise training program is specific and targeted to the patients' primary underlying problems known to establish the primary goal of interest. Moreover, outcome testing is performed in activity specific mode.	The intervention must be aligned with the aim. If the aim is to increase cardiovascular fitness, aerobic training should be used and not primarily strength training. This principle is not met if, for example, the efficacy of a walking intervention is evaluated with a cycling test.
**Individuality:** Each individual will respond uniquely to the same training stimulus. Individuality refers to the modification of training to account for an individual's unique capacity and response to training.	A rationale should be provided on how the needs and abilities of any patient are translated in adaptations in individual training programs. Preferably, the training is tailored based on individual training response.	Exercise is prescribed based on workloads corresponding to a percent of peak workload (watt, speed, heart rate) obtained at baseline testing or intra-intervention tests. Also, day-to-day variations in load are based upon individual training response and not a generic rule.
**Initial values:** Individuals with low initials values in the outcome of interest, will show greater improvements compared to those with higher levels.	The program is individually prescribed based on the results of each participant's baseline fitness assessment. Initial values should be obtained with an appropriate exercise test.	Participants with low levels of, e.g., endurance, will be more likely to see a significant change than those with higher endurance levels. This principle is met if, for example, training load (frequency, duration, or intensity) is based upon initial values.

For peer review only - http://bmjopen.bmj.com/site/about/guidelines.xhtml

**Periodization:** Periodization is characterized by a division of training into periods with specific goals and defined by the variation in training variables such as intensity, duration and frequency.	Rationale is provided for the systematic planning of training periods. Description of manipulation of different training components, e.g. intensity, volume, frequency or recovery.	Example of linear periodization description: "For the first 4 weeks, the training target was 3 series of 6 minutes at 50% of pretraining maximum work rate. Intensity was increased with 5% when subjects completed all 3 series of cycling. When 3 series of 6 minutes cycling were again achieved, loads were increased. After 4 weeks of training, Wmax was reassessed and used for the remaining 6 weeks. Subjects performed five series of 3 minutes using 80% of the new Wmax."
**Reversibility:** Adaptations to exercise training is reversible, meaning that adaptations will return to baseline (detraining) if the training stimulus is removed.	A rationale is provided on how temporarily discontinuation is taken up in the training plan.	Interruptions in the intervention, due to illness, exacerbations or other reasons, must be considered in terms of return-to-training and how the training is recommenced.
The principles of exercise training[26 27 33 34] assessed in this systematic review.		

We have chosen to not include the principle *diminishing returns* in our critical appraisal. We argue that his principle, which states *that the expected degree of improvement in fitness decreases as individuals become more fit, thereby increasing the effort required for further improvements*, is highly difficult to distinctly judge as "yes" or "no", separately from other principles, specifically progression and overload. For example, if both progression and overload is applied, we believe diminishing returns is considered.

For peer review only - http://bmjopen.bmj.com/site/about/guidelines.xhtml

For peer review only

Training variables that will be extracted (extended version of CERT item 13):

- Program duration (weeks)
- Program frequency (sessions/week)
- Exercise intensity (i.e., Watts or km/h)
- Work-phase duration (minutes: seconds)
- Rest periods between series (minutes: seconds): Only applicable for interval training with rest periods, or continuous training were rest periods are allowed.
- Type of rest between series, if applicable (active, passive)
- Intensity of active rest between series, if applicable (i.e., Watts or km/h)
- Rest periods between exercises (minutes: seconds), applicable on rest periods between exercises which are part of the training, including other components than the aerobic training
- Sequence of exercises, if applicable

Outcomes that will be extracted (reported as yes or no and corresponding data):

Primary outcomes

- 6-minute walking distance (6MWD, meters)
- Incremental shuttle walk test (ISWT, meters)
- Endurance shuttle walk test (ESWT, time)
- Peak work rate (watts) or VO_2max (ml/min/kg) during cardiopulmonary exercise test (CPET)
- Constant work-rate test (time, minutes: seconds)

Secondary outcomes

- Short physical performance battery (SPPB, score)
- Sit-to-stand tests: 5-time sit-to-stand, 30-seconds sit-to-stand or 1-minute sit-to-stand (time or reps)
- Timed-up and go test (TUG; sec)
- Stair climb power test (Watt)
- Steep ramp test (Watt)

We consider outcomes related to cardiorespiratory capacity and exercise tolerance primary given that these are vital objectives in international COPD treatment guidelines [3,29,35].

Risk of bias and study quality assessment

The risk of bias and reporting in eligible studies will be assessed independently by two reviewers. Disagreements will be resolved by consensus or including a third reviewer. No study will be excluded due to high risk of bias, but sensitivity analyses excluding poor-quality studies will be performed. The risk of bias assessment applies to our secondary objective describe in this protocol. Thus, the risk of bias assessment will be presented along with the results regarding the secondary objective.

To assess risk of bias, we will use the TESTEX (Tool for the assESsment of sTudy quality and bias in EXercise) tool. TESTEX[36] is specifically designed for evaluating the risk of bias and reporting in exercise training studies. The TESTEX contains 12 measures: five on study quality and seven on study reporting. A total of 15 points can be provided, where higher ratings mean higher quality (lower risk of bias). While no validated cut-off scores are provided for TESTEX, we will categorize the studies according to the average score as "high quality" (≥12 points), "good quality" (7 to 11 points), and "low quality" (≤6 points) as previously described[37].

For peer review only

For peer review only - http://bmjopen.bmj.com/site/about/guidelines.xhtml

Each study will be independently assessed by two reviewers, and discrepancies will be resolved through discussion or consultation with a third reviewer if needed. We will provide a detailed account of the risk of bias for each study, contributing to the overall assessment of the validity and reliability of the evidence. This evaluation will aid in identifying potential sources of bias and in interpreting the findings of the included studies in the context of their methodological strengths and limitations.

Synthesis and quality appraisal of training principles and quality of reporting

Quality appraisal related to our primary objective is performed independently by two reviewers, who are not involved in the included studies. A third reviewer will resolve disagreements and a majority rule will be used.

Given the nature of the data collected for our primary objective, no meta-analysis will be performed. Instead, we will synthesise the data as described below. The synthesis will be guided by relevant items from the PRISMA and synthesis without meta-analysis (SWiM) reporting guidelines. The SWiM[38] guideline is a nine-item checklist to promote transparent reporting of systematic reviews.

The variables listed under participant characteristics, aerobic training intervention characteristics, training principles and training variables will be rated as: "1" (reported), "0" (not reported), "not clear", "insufficient" or "not applicable (NA)".

The sum scores of training principles (up to eight points) and training variables (up to nine points) reported per study will be calculated. Training variables will be corrected for the number of NAs, since different numbers of variables might apply to different studies. The count of exercise training principles met within studies will be presented. The number and percentage of studies complying with all training principles and all relevant training variables will be calculated.

We will also critically appraise the included studies using the CERT checklist[15]. Similarly, each of the 19 items (16 items where items no. 7, 14 and 16 are divided into "a" and "b") in the CERT checklist will be reported as either "1" (reported), "0" (not reported), "not clear", or "not applicable". As such, studies will be provided with 0-19 points on CERT-compliance. In addition, the items "objective of aerobic training intervention", "place of aerobic training intervention", and "attendance" as listed under intervention characteristics will also be scored (0-3 points). Individual scores per paper will be presented in a supplementary material while a summary score per variable is presented within the main text. The number and percentage of studies complying per CERT item will also be calculated. Tables will be used to present these data descriptively.

Publication details, reporting of participant and intervention characteristics will be narratively synthesised and presented using descriptive statistics to synthesise complete reporting. Summary scores will also be provided for participant characteristics.

Meta-analysis

For our second objective, meta-analyses will be conducted to synthesise the efficacy of aerobic training interventions on selected outcomes. We are specifically interested in analysing the effects of application of training principles and complete reporting, as described under "Subgroup analysis and investigation of heterogeneity ".

Meta-analyses will be undertaken where meaningful; if the participants, type of intervention and study design are homogenous enough to warrant pooling. We anticipate only continuous data to be reported for the outcomes of interest. Continuous data will be analysed as the mean difference (MD) where outcome data are reported on a uniform scale or standardized mean difference (SMD) when different metrics are used but deemed clinically homogenous. Meta-analyses will be based on change from

For peer review only - http://bmjopen.bmj.com/site/about/guidelines.xhtml

baseline rather than post-intervention values when available. Data extraction, and any needed imputations or will be done according to recommendations in Cochrane Handbook for Systematic Reviews of Interventions Version 6.4. If needed, additional information from the study authors will be sought using e-mail. A random-effects model will be used to allow for random error and inter-study variability.

We will use per-protocol analyses as the effect of interest, where they are reported. If only intention-to-treat data is reported, this will be used for analysis. If both per-protocol and intention-to-treat data are reported, the intention-to-treat data will be used in a sensitivity analysis. Where multiple trial arms are reported in a single study, we will include only relevant ones. If applicable, we will combine intervention arms or reported subgroups as per the recommendations in Cochrane Handbook for Systematic Reviews of Interventions Version 6.4.

Assessment of heterogeneity

Studies included in the review are likely to be methodologically diverse. As such, heterogeneity in the results is to be expected. We will use the I^2 and τ^2 statistics to measure heterogeneity between the studies in each analysis. If we identify substantial heterogeneity ($I^2 \geq 50\%$), we will report it and explore the possible causes by prespecified subgroup analysis. The importance of the I^2 statistic will be interpreted together with the confidence interval and p-value from the $\chi 2$ statistic, as well as the magnitude and direction of effect as described in the in Cochrane Handbook for Systematic Reviews of Interventions Version 6.4.

Assessment of reporting biases

Publication bias threatens the validity of a review's findings. To address this, if we can pool more than 10 studies, we will create and examine the asymmetry of a funnel plot and perform Egger's test to explore possible small study and publication biases. The Funnel plot provides visual aid for detection of publication bias, indicating whether there is a lack of studies reporting negative or insignificant results.

To further investigate and adjust for publication bias, we will employ the trim-and-fill method[39]. This method is designed to identify and correct for asymmetries in the funnel plot that suggest the presence of publication bias. In the event of funnel plot asymmetry, we will use the trim-and-fill method to impute ostensibly missing effect sizes as a sensitivity analysis. Grading of Recommendations Assessment, Development, and Evaluation (GRADE) is used for assessments of the quality of the evidence.

Subgroup analysis and investigation of heterogeneity

We plan to conduct subgroup analyses to investigate the effect of application of training principles and complete reporting on intervention efficacy:

- Applying relevant principles versus not applying relevant principles: This subgroup analysis will be performed per principle.
- Complete or almost complete reporting of training variables (defined as reporting $\geq$85% of relevant variables, e.g., eight out of nine variables) versus non-complete reporting of training variables (defined as reporting <85% relevant variables)
- High versus low compliance with CERT items. No accepted cut-offs exist for compliance to the CERT. Therefore, a post-hoc decision will be made on which cut-off to use to enable an adequate comparison with reasonable subgroups.
- Studies published after versus before publication of CERT.

For peer review only

For peer review only - http://bmjopen.bmj.com/site/about/guidelines.xhtml

Other planned subgroup analyses, not related to training principles or complete reporting but important effect modifiers, which will be performed if an adequate number of studies per analysis is available:

- Sex.
- Exercise intensity.
- Duration of intervention (≥15 sessions compared with <15 sessions).
- Severity of COPD (GOLD A and B versus C and D or E).

Additional sub-group analyses may be added if an adequate number of studies per analysis is available. Any sub-analysis that is not pre-specified will be clearly presented as such and presented in a supplementary material.

To further investigate if methodological diversity moderates the effect on the selected outcomes, a meta-regression will be performed. A meta-regression is useful when there is a wide range of values in moderator variables but few studies with the exact same value for that moderator. We expect a wide range in moderator variables such as number of CERT items reported, or training principles applied.

We will employ a random-effects univariate meta-regression to examine the relationship between the extent of complete reporting, application of training principles and change in outcomes[40].

The dependent variable in the meta-regression will be the mean difference in a continuous outcome (for example, change in 6MWD). We will examine how this effect estimate is influenced by explanatory variables, such as number of applied training principles or compliance with CERT (number of items). As such, the meta-regression model aims to predict the study's effect size, based on characteristics of the studies.

Sensitivity analysis

We plan to carry out the following sensitivity analyses, removing the following from the primary outcome analyses to examine whether findings are robust to potentially influential decisions:

- Trials judged in the quality assessment to be of low quality.
- Trials with low or no compliance to training principles, defined as applying zero, one or two (out of eight) training principles.
- Trials reporting a low number of training variables (defined as reporting 33% or less of relevant variables).
- Trials with low compliance with the CERT checklist. No generally accepted cut-offs exist for compliance to the CERT. Therefore, a post-hoc decision will be made on which cut-off to use to enable an adequate sensitivity analysis.

We will compare results from a fixed-effect model versus a random-effect model. If we impute SD using correlation coefficients in the synthesis, sensitivity analyses will be performed with different values of the correlation coefficient to determine if the overall results are robust to the imputed correlation coefficients[40]. The sensitivity analyses will be presented in a summary table. Other suitable sensitivity analyses, as identified during the review process, may also be performed, and reported.

Assessment of the certainty of the evidence

We will employ GRADE approach to assess the certainty of the body of evidence related to the included primary and secondary outcomes. The GRADE approach includes assessing evidence across several key

For peer review only

For peer review only - http://bmjopen.bmj.com/site/about/guidelines.xhtml

dimensions: Risk of bias, imprecision, inconsistency, indirectness, and publication bias. Thus, the quality of the evidence will be graded as 'high', 'moderate', 'low' or 'very low'. We will use the methods and recommendations described in the Cochrane Handbook for Systematic Reviews of Interventions V.6.4 using the GRADEpro software.

Patient and public involvement

None.

ETHICS AND DISSEMINATION

As this study is a systematic review, ethical approval is not required

The systematic review will be submitted for publication in a peer-reviewed journal. We also intend to present the findings at national and international conferences and inform patient organisations.

We will conduct the review according to this protocol and justify any deviations in a specific section of the systematic review results report.

DISCUSSION

This systematic review is designed to critically assess the application of exercise training principles and the completeness of reporting in RCTs with an aerobic training intervention in the COPD literature. This is, to our knowledge, the first systematic review addressing this topic in COPD trials.

The findings of this systematic review are expected to indicate shortcomings in aerobic training interventions and reporting of RCTs in COPD. Based on the findings, we will provide recommendations for future work to enhance the quality of exercise prescription as well as study quality and reporting practices. Identified limitations or strengths in the included studies could inform future guidelines revisions. This, in turn, might lead to more tailored and effective exercise training in pulmonary rehabilitation.

Registration of the systematic review and publication of this protocol promotes transparency in the review process. It also minimises the risk of duplication of similar reviews. We strive for a high methodological standard by adhering to PRISMA-P, and SWiM guidelines. This review is not without limitations. The exclusion of literature written in other languages than English might leave relevant literature out of the systematic review. For this reason, we will report the number of publications that are excluded due to this reason.

For peer review only

For peer review only - http://bmjopen.bmj.com/site/about/guidelines.xhtml

Contributors

JJ contributed to the design of the study and the protocol, developed the data synthesis and meta-analysis, and wrote the initial version of the manuscript. AS contributed to the design of the study and the protocol, developed the search strategy, data extraction and data synthesis process. JJ and AS contributed equally as co-first authors. HvH contributed to conceptualising the study, the design of the protocol and reviewed the protocol critically for important intellectual content. JDB contributed to the design of the protocol, developed the data synthesis and meta-analysis, and reviewed the protocol critically for important intellectual content. AN contributed to conceptualising the study, the design of the protocol, developed the data synthesis and meta-analysis, and reviewed the protocol critically for important intellectual content. PK conceptualised the study, developed the data synthesis and meta-analysis, as the guarantor for the protocol and reviewed the protocol critically for important intellectual content. AN and PK contributed equally as co-senior authors. All authors read, approved, and contributed to the final written manuscript.

Funding

This work is undertaken as part of JJ's PhD work, funded by the Swedish Research Council (Vetenskapsrådet), grant number: 2020-01296. AS was financially supported by Lung Foundation [#5.1.18.232], Netherlands. Funders have had no role in the design of this protocol or in the systematic review; nor in the decision to report and publish the results. Non-financial support has been provided by the library at Radboud University, in developing the search strategy.

Competing interests

None declared.

Patient consent

Not applicable.

For peer review only

References

1. World Health Organization. The top 10 causes of death World Health Organization; 2020 [cited 2021. Available from: https://www.who.int/news-room/fact-sheets/detail/the-top-10-causes-of-death accessed 2021-09-07 2021.
2. (GOLD) GIfCOLD. The Global Strategy for Diagnosis, Management and Prevention of COPD, 2024 report, 2023.
3. Spruit MA, Singh SJ, Garvey C, et al. An official American Thoracic Society/European Respiratory Society statement: key concepts and advances in pulmonary rehabilitation. *Am J Respir Crit Care Med* 2013;188(8):e13-64. doi: 10.1164/rccm.201309-1634ST [published Online First: 2013/10/17]
4. Gloeckl R, Zwick RH, Fürlinger U, et al. Prescribing and adjusting exercise training in chronic respiratory diseases - Expert-based practical recommendations. *Pulmonology* 2023;29(4):306-14. doi: 10.1016/j.pulmoe.2022.09.004 [published Online First: 20221020]
5. Rochester CL, Alison JA, Carlin B, et al. Pulmonary Rehabilitation for Adults with Chronic Respiratory Disease: An Official American Thoracic Society Clinical Practice Guideline. *Am J Respir Crit Care Med* 2023;208(4):e7-e26. doi: 10.1164/rccm.202306-1066ST
6. Ward TJC, Plumptre CD, Dolmage TE, et al. Change in $\dot{V}$O2peak in Response to Aerobic Exercise Training and the Relationship With Exercise Prescription in People With COPD: A Systematic Review and Meta-analysis. *Chest* 2020;158(1):131-44. doi: https://doi.org/10.1016/j.chest.2020.01.053
7. Higashimoto Y, Ando M, Sano A, et al. Effect of pulmonary rehabilitation programs including lower limb endurance training on dyspnea in stable COPD: A systematic review and meta-analysis. *Respir Investig* 2020;58(5):355-66. doi: 10.1016/j.resinv.2020.05.010 [published Online First: 20200711]
8. Ryrsø CK, Godtfredsen NS, Kofod LM, et al. Lower mortality after early supervised pulmonary rehabilitation following COPD-exacerbations: a systematic review and meta-analysis. *BMC Pulm Med* 2018;18(1):154. doi: 10.1186/s12890-018-0718-1
9. Paneroni M, Simonelli C, Vitacca M, et al. Aerobic Exercise Training in Very Severe Chronic Obstructive Pulmonary Disease: A Systematic Review and Meta-Analysis. *Am J Phys Med Rehabil* 2017;96(8):541-48. doi: 10.1097/phm.0000000000000667
10. Gloeckl R, Marinov B, Pitta F. Practical recommendations for exercise training in patients with COPD. *Eur Respir Rev* 2013;22(128):178-86. doi: 10.1183/09059180.00000513
11. Begg C, Cho M, Eastwood S, et al. Improving the quality of reporting of randomized controlled trials. The CONSORT statement. *JAMA* 1996;276(8):637-9. doi: 10.1001/jama.276.8.637
12. Schulz KF, Altman DG, Moher D. CONSORT 2010 statement: updated guidelines for reporting parallel group randomised trials. *BMJ* 2010;340:c332. doi: 10.1136/bmj.c332 [published Online First: 2010/03/25]
13. Glasziou P, Altman DG, Bossuyt P, et al. Reducing waste from incomplete or unusable reports of biomedical research. *Lancet* 2014;383(9913):267-76. doi: 10.1016/s0140-6736(13)62228-x [published Online First: 20140108]
14. Arienti C, Armijo-Olivo S, Minozzi S, et al. Methodological Issues in Rehabilitation Research: A Scoping Review. *Arch Phys Med Rehabil* 2021;102(8):1614-22.e14. doi: 10.1016/j.apmr.2021.04.006 [published Online First: 20210511]
15. Slade SC, Dionne CE, Underwood M, et al. Consensus on Exercise Reporting Template (CERT): Explanation and Elaboration Statement. *Br J Sports Med* 2016;50(23):1428-37. doi: 10.1136/bjsports-2016-096651 [published Online First: 2016/10/07]
16. Mesquida C, Murphy J, Lakens D, et al. Replication concerns in sports and exercise science: a narrative review of selected methodological issues in the field. *Royal Society Open Science* 2022;9(12):220946. doi: 10.1098/rsos.220946

For peer review only

For peer review only - http://bmjopen.bmj.com/site/about/guidelines.xhtml

17. Hoogeboom TJ, Kousemaker MC, van Meeteren NL, et al. i-CONTENT tool for assessing therapeutic quality of exercise programs employed in randomised clinical trials. *Br J Sports Med* 2020 doi: 10.1136/bjsports-2019-101630 [published Online First: 2020/11/05]
18. Moecke DP, Zhu K, Gill J, et al. A systematic review of exercise studies for individuals hospitalized with an acute exacerbation of chronic obstructive pulmonary disease: Focus on the principles of exercise training. *Chron Respir Dis* 2023;20:14799731231215363. doi: 10.1177/14799731231215363
19. Westra B, de Wolf S, Bij de Vaate E, et al. Quality of resistance training description in COPD trials: study protocol for a systematic review. *BMJ open* 2019;9(1):e025030. doi: https://dx.doi.org/10.1136/bmjopen-2018-025030
20. Holden S, Rathleff MS, Jensen MB, et al. How can we implement exercise therapy for patellofemoral pain if we don't know what was prescribed? A systematic review. *Br J Sports Med* 2018;52(6):385. doi: 10.1136/bjsports-2017-097547 [published Online First: 20171030]
21. Jo D, Del Bel MJ, McEwen D, et al. A study of the description of exercise programs evaluated in randomized controlled trials involving people with fibromyalgia using different reporting tools, and validity of the tools related to pain relief. *Clin Rehabil* 2019;33(3):557-63. doi: 10.1177/0269215518815931 [published Online First: 2018/12/06]
22. Medysky ME, Bland KA, Neil-Sztramko SE, et al. Attention to the Principles of Exercise Training in Exercise Studies of Persons With Lung Cancer: A Systematic Review. *J Aging Phys Act* 2021:1-11. doi: 10.1123/japa.2020-0269 [published Online First: 2021/05/09]
23. Neil-Sztramko SE, Medysky ME, Campbell KL, et al. Attention to the principles of exercise training in exercise studies on prostate cancer survivors: a systematic review. *BMC Cancer* 2019;19(1):321. doi: 10.1186/s12885-019-5520-9 [published Online First: 2019/04/07]
24. Neil-Sztramko SE, Winters-Stone KM, Bland KA, et al. Updated systematic review of exercise studies in breast cancer survivors: attention to the principles of exercise training. *Br J Sports Med* 2019;53(8):504-12. doi: 10.1136/bjsports-2017-098389 [published Online First: 2017/11/23]
25. Hoffman J. Physiological Aspects of Sport Training and Performance. Champaign: IL: Human Kinetics 2002.
26. Kasper K. Sports Training Principles. *Curr Sports Med Rep* 2019;18(4)
27. Campbell KL, Neil SE, Winters-Stone KM. Review of exercise studies in breast cancer survivors: attention to principles of exercise training. *Br J Sports Med* 2012;46(13):909-16. doi: 10.1136/bjsm.2010.082719

10.1136/bjsports-2010-082719 [published Online First: 2012/09/26]
28. Thomas JH, Martijn CK, Nico LUvM, et al. i-CONTENT tool for assessing therapeutic quality of exercise programs employed in randomised clinical trials. *Br J Sports Med* 2021;55(20):1153. doi: 10.1136/bjsports-2019-101630
29. McCarthy B, Casey D, Devane D, et al. Pulmonary rehabilitation for chronic obstructive pulmonary disease. *Cochrane Database Syst Rev* 2015(2):Cd003793. doi: 10.1002/14651858.CD003793.pub3 [published Online First: 2015/02/24]
30. Page MJ, McKenzie JE, Bossuyt PM, et al. The PRISMA 2020 statement: an updated guideline for reporting systematic reviews. *BMJ* 2021;372:n71. doi: 10.1136/bmj.n71
31. Nici L, Donner C, Wouters E, et al. American Thoracic Society/European Respiratory Society statement on pulmonary rehabilitation. *Am J Respir Crit Care Med* 2006;173(12):1390-413. doi: 10.1164/rccm.200508-1211ST [published Online First: 2006/06/09]
32. Ouzzani M, Hammady H, Fedorowicz Z, et al. Rayyan—a web and mobile app for systematic reviews. *Systematic Reviews* 2016;5(1):210. doi: 10.1186/s13643-016-0384-4
33. Bland KA, Neil-Sztramko SE, Zadravec K, et al. Attention to principles of exercise training: an updated systematic review of randomized controlled trials in cancers other than breast and prostate. *BMC Cancer* 2021;21(1):1179. doi: 10.1186/s12885-021-08701-y

For peer review only - http://bmjopen.bmj.com/site/about/guidelines.xhtml

34. Winters-Stone KM, Neil SE, Campbell KL. Attention to principles of exercise training: a review of exercise studies for survivors of cancers other than breast. *Br J Sports Med* 2014;48(12):987-95. doi: 10.1136/bjsports-2012-091732 [published Online First: 2013/01/08]
35. Nyberg A, Probst V, Vaes AW. Assessments. In: Holland AE, Corso SD, Spruit MA, eds. Pulmonary Rehabilitation: European Respiratory Society 2021:360.
36. Smart NA, Waldron M, Ismail H, et al. Validation of a new tool for the assessment of study quality and reporting in exercise training studies: TESTEX. *Int J Evid Based Healthc* 2015;13(1):9-18. doi: 10.1097/xeb.0000000000000020
37. Stefanakis M, Batalik L, Antoniou V, et al. Safety of home-based cardiac rehabilitation: A systematic review. *Heart Lung* 2022;55:117-26. doi: https://doi.org/10.1016/j.hrtlng.2022.04.016
38. Campbell M, McKenzie JE, Sowden A, et al. Synthesis without meta-analysis (SWiM) in systematic reviews: reporting guideline. *BMJ* 2020;368:l6890. doi: 10.1136/bmj.l6890
39. Duval S, Tweedie R. Trim and Fill: A Simple Funnel-Plot-Based Method of Testing and Adjusting for Publication Bias in Meta-Analysis. *Biometrics* 2000;56(2):455-63.
40. Higgins JPT TJ, Chandler J, Cumpston M, Li T, Page MJ, Welch VA (editors). Cochrane Handbook for Systematic Reviews of Interventions version 6.2 (updated February 2021): Cochrane, 2021, 2021.

For peer review only

For peer review only - http://bmjopen.bmj.com/site/about/guidelines.xhtml

Reporting checklist for protocol of a systematic review and meta analysis.

Based on the PRISMA-P guidelines.

Instructions to authors

Complete this checklist by entering the page numbers from your manuscript where readers will find each of the items listed below.

Your article may not currently address all the items on the checklist. Please modify your text to include the missing information. If you are certain that an item does not apply, please write "n/a" and provide a short explanation.

Upload your completed checklist as an extra file when you submit to a journal.

In your methods section, say that you used the PRISMA-Preporting guidelines, and cite them as:

Moher D, Shamseer L, Clarke M, Ghersi D, Liberati A, Petticrew M, Shekelle P, Stewart LA. Preferred Reporting Items for Systematic Review and Meta-Analysis Protocols (PRISMA-P) 2015 statement. Syst Rev. 2015;4(1):1.

		Reporting Item	Page Number
**Title**			
Identification	#1a	Identify the report as a protocol of a systematic review	1
Update	#1b	If the protocol is for an update of a previous systematic review, identify as such	NA
**Registration**			
	#2	If registered, provide the name of the registry (such as PROSPERO) and registration number	2
**Authors**			
Contact	#3a	Provide name, institutional affiliation, e-mail address of all protocol authors; provide physical mailing address of corresponding author	1
Contribution	#3b	Describe contributions of protocol authors and identify the	17

For peer review only

		guarantor of the review	
**Amendments**			
	#4	If the protocol represents an amendment of a previously completed or published protocol, identify as such and list changes; otherwise, state plan for documenting important protocol amendments	16
**Support**			
Sources	#5a	Indicate sources of financial or other support for the review	17
Sponsor	#5b	Provide name for the review funder and / or sponsor	NA
Role of sponsor or funder	#5c	Describe roles of funder(s), sponsor(s), and / or institution(s), if any, in developing the protocol	17
**Introduction**			
Rationale	#6	Describe the rationale for the review in the context of what is already known	4-5
Objectives	#7	Provide an explicit statement of the question(s) the review will address with reference to participants, interventions, comparators, and outcomes (PICO)	5
**Methods**			
Eligibility criteria	#8	Specify the study characteristics (such as PICO, study design, setting, time frame) and report characteristics (such as years considered, language, publication status) to be used as criteria for eligibility for the review	5-6
Information sources	#9	Describe all intended information sources (such as electronic databases, contact with study authors, trial registers or other grey literature sources) with planned dates of coverage	6-7
Search strategy	#10	Present draft of search strategy to be used for at least one electronic database, including planned limits, such that it could be repeated	7 and appendix
Study records - data management	#11a	Describe the mechanism(s) that will be used to manage records and data throughout the review	7-8

For peer review only

Study records - selection process	#11b	State the process that will be used for selecting studies (such as two independent reviewers) through each phase of the review (that is, screening, eligibility and inclusion in meta-analysis)	7
Study records - data collection process	#11c	Describe planned method of extracting data from reports (such as piloting forms, done independently, in duplicate), any processes for obtaining and confirming data from investigators	8-12
Data items	#12	List and define all variables for which data will be sought (such as PICO items, funding sources), any pre-planned data assumptions and simplifications	8-12
Outcomes and prioritization	#13	List and define all outcomes for which data will be sought, including prioritization of main and additional outcomes, with rationale	12
Risk of bias in individual studies	#14	Describe anticipated methods for assessing risk of bias of individual studies, including whether this will be done at the outcome or study level, or both; state how this information will be used in data synthesis	12-13 + 15
Data synthesis	#15a	Describe criteria under which study data will be quantitatively synthesised	14-15
Data synthesis	#15b	If data are appropriate for quantitative synthesis, describe planned summary measures, methods of handling data and methods of combining data from studies, including any planned exploration of consistency (such as I2, Kendall's τ)	14-15
Data synthesis	#15c	Describe any proposed additional analyses (such as sensitivity or subgroup analyses, meta-regression)	14-15
Data synthesis	#15d	If quantitative synthesis is not appropriate, describe the type of summary planned	13-14
Meta-bias(es)	#16	Specify any planned assessment of meta-bias(es) (such as publication bias across studies, selective reporting within studies)	14
Confidence in cumulative evidence	#17	Describe how the strength of the body of evidence will be assessed (such as GRADE)	15-16

For peer review only

Notes:

- 10: 8 and appendix The PRISMA-P elaboration and explanation paper is distributed under the terms of the Creative Commons Attribution License CC-BY. This checklist was completed on 12. January 2024 using https://www.goodreports.org/, a tool made by the EQUATOR Network in collaboration with Penelope.ai

For peer review only

Online appendix 2. Search strategies

The number within brackets after every search terms is the number of hits for that search.

1. Search strategy for PubMed

Date: 27-05-2021

#1:

"Pulmonary Disease, Chronic Obstructive"[Mesh] OR Chronic Obstructive Pulmonary Disease* [tiab] OR COPD [tiab] OR COAD [tiab] OR Chronic Obstructive Airway Disease* [tiab] OR Chronic Obstructive Lung Disease* [tiab] OR chronic airflow obstruction* [tiab] OR chronic airflow limitation*[tiab] OR Emphysema[tiab] OR Chronic bronchitis[tiab] OR chronic obstructive bronchitis[tiab] OR chronic obstructive bronchopulmonary disease*[tiab] OR chronic obstructive lung disorder*[tiab] OR chronic obstructive pulmonary disorder*[tiab] OR chronic obstructive respiratory disease*[tiab] OR lung chronic obstructive disease*[tiab] (111 965)

#2:

"Pulmonary Disease, Chronic Obstructive/rehabilitation"[Mesh] OR "Rehabilitation"[Mesh:NoExp] OR Exercise Therapy[Mesh] OR "Exercise"[Mesh] OR "Bicycling"[Mesh] OR Aerobic training* [tiab] OR aerobic exercise*[tiab] OR exercise training*[tiab] OR rehabilitation[tiab] OR Exercise Therap*[tiab] OR Endurance Training*[tiab] OR Walking[tiab] OR swimming[tiab] OR rowing[tiab] OR endurance exercise*[tiab] OR interval training*[tiab] OR cycling[tiab] OR bicycling[tiab] OR training*[tiab] OR hit[tiab] (941 554)

#3: #1 AND #2 (9 021)

#4: #1 AND #2 Filters: English (7 931)

#5: #1 AND #2 Filters: from 2007 – 3000/12/12 (6 344)

#6: #1 AND #2 Filters: English, from 2007 – 3000/12/12 (5 828)

Total hits: 5 828

For peer review only

Begränsad delning

2. Search strategy for Embase

Date: 27-05-2021

1. *chronic obstructive lung disease/ or *lung emphysema/ or *chronic bronchitis/ or (Chronic Obstructive Pulmonary Disease* or COPD or COAD or Chronic Obstructive Airway Disease* or Chronic Obstructive Lung Disease* or chronic airflow obstruction* or chronic airflow limitation* or Emphysema or Chronic bronchitis or chronic obstructive bronchitis or chronic obstructive bronchopulmonary disease* or chronic obstructive lung disorder* or chronic obstructive pulmonary disorder* or chronic obstructive respiratory disease* or lung chronic obstructive disease*).ti,ab,kw. (170 046)

2. chronic obstructive lung disease/rh or lung emphysema/rh or chronic bronchitis/rh or exercise/ or aerobic exercise/ or endurance training/ or high intensity interval training/ or training/ or rehabilitation/ or pulmonary rehabilitation/ or kinesiotherapy/ or walking/ or swimming/ or rowing/ or cycling/ or (Aerobic training* or aerobic exercise* or exercise training* or rehabilitat* or Exercise Therap* or Endurance Training* or Walking or swimming or rowing or endurance exercise* or interval training* or cycling or bicycling or training* or hit).ti,ab,kw. (1 349 026)

3. 1 and 2 (18 989)

4. limit 3 to english language (16 992)

5. limit 3 to yr="2007 -Current" (14 531)

6. limit 3 to (english language and yr="2007 -Current") (13 740)

7. limit 6 to conference abstract status (6 130)

8. 6 not 7 (7 610)

Total hits: 7 610

For peer review only

Begränsad delning

3. Search strategy for Cinahl

Date: 27-05-2021

S1. (MH "Pulmonary Disease, Chronic Obstructive+") OR (MH "Emphysema") OR TI ("Chronic Obstructive Pulmonary Disease*" OR COPD OR COAD OR "Chronic Obstructive Airway Disease*" OR "Chronic Obstructive Lung Disease*" OR "chronic airflow obstruction*" OR "chronic airflow limitation*" OR Emphysema OR "Chronic bronchitis" OR "chronic obstructive bronchitis" OR "chronic obstructive bronchopulmonary disease*" OR "chronic obstructive lung disorder*" OR "chronic obstructive pulmonary disorder*" OR "chronic obstructive respiratory disease*" OR "lung chronic obstructive disease*") OR AB ("Chronic Obstructive Pulmonary Disease*" OR COPD OR COAD OR "Chronic Obstructive Airway Disease*" OR "Chronic Obstructive Lung Disease*" OR "chronic airflow obstruction*" OR "chronic airflow limitation*" OR Emphysema OR "Chronic bronchitis" OR "chronic obstructive bronchitis" OR "chronic obstructive bronchopulmonary disease*" OR "chronic obstructive lung disorder*" OR "chronic obstructive pulmonary disorder*" OR "chronic obstructive respiratory disease*" OR "lung chronic obstructive disease*") (33 553)

Expanders: Apply equivalent subjects

Search modes: Boolean/Phrase

S2. (MH "Pulmonary Disease, Chronic Obstructive+/RH") OR (MH "Emphysema/RH") OR (MH "Exercise+") OR (MH "Physical Therapy+") OR (MH "Rehabilitation") OR (MH "Rehabilitation, Pulmonary+") OR (MH "Swimming") OR (MH "Rowing") OR (MH "Cycling") OR TI ("Aerobic training*" OR "aerobic exercise*" OR "exercise training*" OR rehabilitat* OR "Exercise Therap*" OR "Endurance Training*" OR Walking OR swimming OR rowing OR "endurance exercise*" OR "interval training*" OR cycling OR bicycling OR training* OR hit) OR AB ("Aerobic training*" OR "aerobic exercise*" OR "exercise training*" OR rehabilitat* OR "Exercise Therap*" OR "Endurance Training*" OR Walking OR swimming OR rowing OR "endurance exercise*" OR "interval training*" OR cycling OR bicycling OR training* OR hit) (511 922)

Expanders: Apply equivalent subjects

Search modes: Boolean/Phrase

S3. S1 AND S2 (4 398)

Expanders: Apply equivalent subjects

Search modes: Boolean/Phrase

S4. S1 AND S2. (4 147)

Expanders: Apply equivalent subjects

Search modes: Boolean/Phrase

Narrow by Language: English

S5. S1 AND S2 (3 344)

Limiters: Published Date: 20070101-20211231

For peer review only

Begränsad delning

Search modes: Boolean/Phrase

Narrow by Language: English

S6. S1 and S2 (3 130)

Limiters: Published Date: 20070101-20211231

Search modes: Boolean/Phrase

Narrow by Language: English

Expanders – Apply equivalent subjects

Total hits: 3 130

4. Cochrane search strategy

Date: 27-05-2021

Expanders: Word variations enabled.

#1: ("Chronic Obstructive Pulmonary Disease" OR COPD OR COAD OR "Chronic Obstructive Airway Disease" OR "Chronic Obstructive Lung Disease" OR "chronic airflow obstruction" OR "chronic airflow limitation" OR Emphysema OR "Chronic bronchitis" OR "chronic obstructive bronchitis" OR "chronic obstructive bronchopulmonary disease" OR "chronic obstructive lung disorder" OR "chronic obstructive pulmonary disorder" OR "chronic obstructive respiratory disease" OR "lung chronic obstructive disease") :ti,ab,kw

#2: ("Aerobic training" OR "aerobic exercise" OR "exercise training" OR rehabilitat* OR "Exercise Therapy" OR "Endurance Training" OR Walking OR swimming OR rowing OR "endurance exercise" OR "interval training" OR cycling OR bicycling OR training OR hit) :ti,ab,kw

#3: #1 AND #2

Cochrane reviews: 38

Trials: 5415

Cochrane protocols: 8, not included

Editorials: 2, not included

Clinical Answers: 2, not included

Total hits: 5453

For peer review only

5. PEDro search strategy

Date: 28-05-2021

#1. Chronic respiratory disease (topic) + aerobic training (abstract and title) (86)

- Limit to systematic reviews and clinical trials (84)
- Other chronic respiratory diseases than COPD excluded (33):
- Remaining hits: 51
- Other languages removed (4)

Total records remaining: 47

For peer review only
